# Prediction of Combined Sorbent and Catalyst Materials for SE-SMR, Using QSPR and Multitask Learning

**DOI:** 10.1021/acs.iecr.2c00971

**Published:** 2022-06-23

**Authors:** Paula Nkulikiyinka, Stuart T. Wagland, Vasilije Manovic, Peter T. Clough

**Affiliations:** Energy and Power Theme, School of Water, Energy and Environment, Cranfield University, Cranfield, Bedfordshire MK43 0AL, U.K.

## Abstract

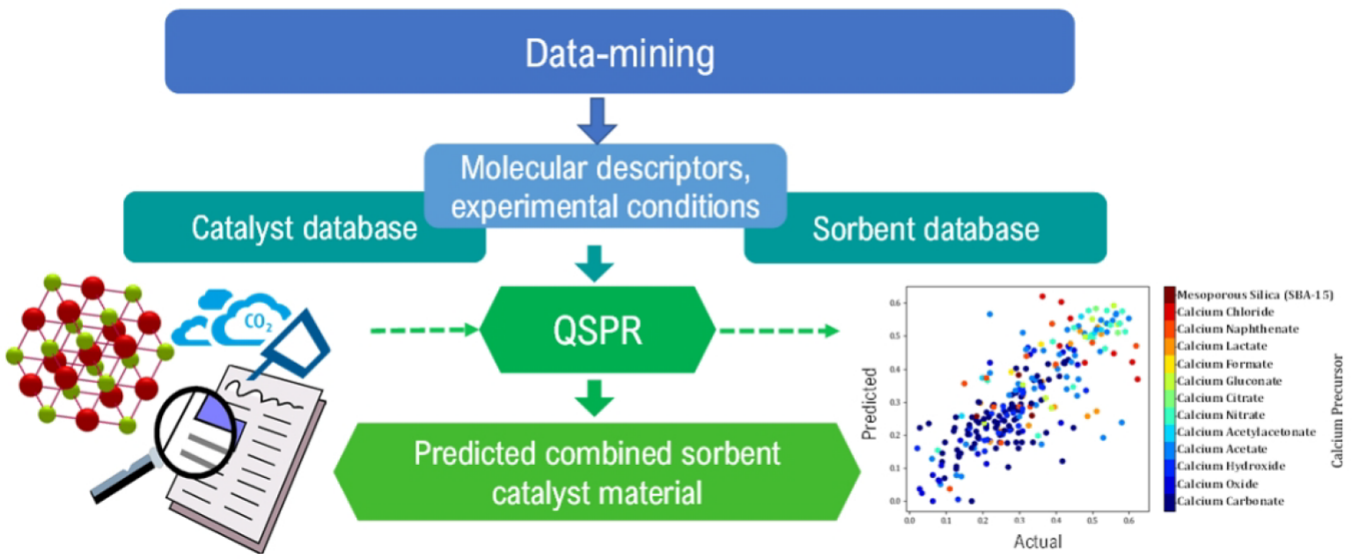

The process of sorption enhanced steam methane reforming (SE-SMR) is an emerging technology for the production of low carbon hydrogen. The development of a suitable catalytic material, as well as a CO_2_ adsorbent with high capture capacity, has slowed the upscaling of this process to date. In this study, to aid the development of a combined sorbent catalyst material (CSCM) for SE-SMR, a novel approach involving quantitative structure–property relationship analysis (QSPR) has been proposed. Through data-mining, two databases have been developed for the prediction of the last cycle capacity (g_CO_2__/g_sorbent_) and methane conversion (%). Multitask learning (MTL) was applied for the prediction of CSCM properties. Patterns in the data of this study have also yielded further insights; colored scatter plots were able to show certain patterns in the input data, as well as suggestions on how to develop an optimal material. With the results from the actual vs predicted plots collated, raw materials and synthesis conditions were proposed that could lead to the development of a CSCM that has good performance with respect to both the last cycle capacity and the methane conversion.

## 1 Introduction

Hydrogen is seen as an attractive energy source for many reasons, including the high potential to reduce carbon emissions by decarbonizing multiple sectors and higher efficiency due to a higher energy density, compared to hydrocarbon fuels such as diesel or gasoline.^1,2^ This gives hydrogen the ability to store a lot more energy for every unit weight of fuel in comparison. Hydrogen production as a fuel source is vital in the race toward the 2050 Net-Zero Target. One of the most promising methods for the future production of low carbon H_2_ is via sorbent enhanced steam methane reforming (SE-SMR),^3^ (reaction 3), which combines the two processes of steam methane reforming (SMR) (reaction 1) and calcium looping (CaL) (reaction 2).
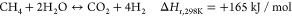
1
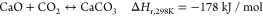
2
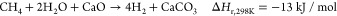
3

In the SE-SMR process, the reforming reaction takes place in the presence of a calcium oxide (CaO)-based sorbent, which allows it to capture in situ the produced CO_2_, which then drives the reaction equilibrium of reaction 1 to the right, leading to higher hydrogen yields. So, while the introduction of a sorbent in the conventional steam reforming process is beneficial for those two reasons, there are also some challenges that come with the use of a sorbent.

First, the selection of sorbent itself poses an issue as the material of choice must process several properties in order for efficient operation, in terms of economics and operating conditions. One of the important requirements of a sorbent in the use of SE-SMR is its carrying capacity over multiple cycles.^4,5^ Other important factors include the following:

- the ability for the adsorbent to be regenerated
- the adsorbent having a high CO_2_ capacity in the kinetic limited regime
- economic viability with a potential export market for the spent material
- having fast reaction kinetics for the absorption and desorption steps
- maintaining adequate mechanical strength after multiple cycles

From early studies on sorbents used in steam methane reforming, further knowledge has been gained on what makes an efficient and appropriate sorbent material. For example, additional factors include durability comparable to the catalysts, to minimize the purge requirements for the spent sorbent;^6^ an adequate pore size distribution with large pore volume in the 50–100 μm range, and active surface of fresh sorbent.^7^ Reaction temperature, carbonation and calcination duration times, atmospheric conditions, sorbent precursor, and sorbent particle size are all key influencers in determining the suitability of a sorbent for SE-SMR. A principal indicator of sorbent performance is the number of reaction cycles and the capacity capability at the last cycle, which essentially suggests the lifetime of a sorbent before replenishment is required.^8^

CaO is a favorable material due to its low cost and wide availability in naturally occurring minerals, for example, limestone and dolomite. Despite calcium-based sorbents being a favored material, a disadvantage of naturally occurring calcium-based materials is the rapid decrease of their CO_2_ uptake capacity with cycle number, due to sintering or pore plugging. Some proposed mechanisms to improve the CO_2_ capture characteristics of limestone, include methods such as hydration or thermal pretreatment.^9^ The choice of the CaO precursor has also been reported to have an effect on the sorption properties of the final synthesized material, with calcium acetate proven to be a high performer.^10^

Another challenge faced in the SE-SMR process is catalyst selection. Catalyst materials used for SE-SMR should resist coke formation and sulfation poisoning, be inactive for side-reactions, maintain the activity at high temperature, and have high mechanical strength, as well as good heat transfer properties.^11^ Preferably, they should also be able to operate at low steam/carbon ratios in order to improve the energy efficiency of the process.^12^ The mechanisms of several catalysts (nickel, rhodium, platinum, ruthenium, and iridium) for SMR reactions were investigated,^13^ for which it was reported that platinum catalysts were most reactive. Nickel oxide has widely been proven to be preferable for use in SE-SMR due to its high catalytic activity, that is, high conversion rate of methane and lower cost compared to rare earth elements.^14−16^ However, nickel does have its disadvantages:^17^

- Nickel nanoclusters (as opposed to a skeletal structure) are prone to sintering from the high temperatures.
- Nickel nanoclusters have a tendency toward coking leading to particle fracturing and active site loss.
- The production of hydrogen-rich gas with a low concentration of CO is a challenge using nickel catalysts, as they are not as active in the water gas shift reaction as other catalysts.

Other factors to consider in catalyst choice include their sensitivity to sulfur poisoning. It is assumed that the natural gas feed will have undergone desulfurization; however, most naturally occurring limestones and dolomites may still contain small quantities of sulfur. Therefore, in the reforming reaction, with enough sulfur present, it can be transferred from the sorbent to the gas phase to quickly poison the nickel reforming catalyst.^18^ The use of metal dispersion and alloying (particularly with platinum, palladium, or ruthenium), are both effective methods to enhance certain properties of catalysts, including resistance to sintering as well as resistance to nickel oxidation, which promotes its activation.^19^ The addition of a metal such as iron, copper, or tin, also has the possibility to improve the catalytic activity.^17^

The concept of a material that possesses both catalytic and adsorbing properties is not a new one and has been of interest for the last 20 years. A combined sorbent catalyst material (CSCM), or a bifunctional material, is a one-particle system that contains a metal for the catalysis, and a sorbent for the in situ CO_2_ sorption. Due to the intimate contact of catalyst and sorbent in the solid, the CSCM particle system enables greater heat transfer and reduced mean path length for mass transfer of the reaction products, which enables a greater conversion efficiency compared to a two-particle system.^20^ Additionally, the overall reactor volume can be reduced by using a sorbent that acts as a support.

There have been different approaches taken to develop and predict the behavior of optimal properties for a suitable CSCM, such as the determination of kinetic and diffusion parameters and computational modeling,^21−25^ as well as material synthesis and experimental studies in a fluidized bed reactor^26^ or fixed bed reactors.^27−29^ The application of CSCM also is not limited to the process of SE-SMR with methane as a carbon source, as there are many studies conducted on CSCM applied in sorption enhanced steam reforming using other raw materials such as glycerol,^30,31^ phenol,^32^ coal,^33^ biomass,^20^ ethanol,^34^ methanol.^35^

The development of an “optimal” CSCM has proven to be a complicated and monotonous task; however, this can be overcome with the application of machine learning, specifically the process of quantitative structure–property relationship analysis (QSPR). This analysis is based on using the molecular structure of a material to predict its physical behavior. QSPR modeling has long been used particularly in the pharmaceutical industry for drug development, and it has recently seen expansion into various physical chemistry fields, including the adsorption of metal organic frameworks.^36−39^ Additionally, the use of QSPR in combination with other computational methods such as DFT or process simulation has also garnered effective prediction within research in the energy field;^40,41^ however, to the best of our knowledge, QSPR has not been applied to the development of a CSCM material for use in SE-SMR.

In this study, we propose a new QSPR machine learning approach for the prediction of a CSCM, through the development of a calcium-based sorbent database and nickel-based catalyst database, consisting of SE-SMR, and SMR experimental data from the literature. The properties of interest used as a measure of the performance for the CSCM, is the methane conversion and the CO_2_ adsorption capacity for the last cycle calcination/carbonation. The individual databases were first developed into QSPR models for the prediction of separate sorbent and catalyst materials, then eventually integrated for the prediction of unseen CSCM materials.

## 2 Methodology

**Figure 1:**
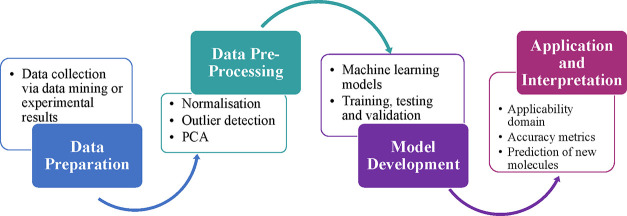
Process flow diagram of the development of QSPR models.

The methodology typically used in QSPR studies consists of six steps that fall into four main categories outlined in Figure 1:

Preparation of data:
  1. The collection or measurement of property data points (here, the last cycle capacity and the methane conversion).
  2. Collection of descriptor data (descriptor data was selected via the OCHEM software. Different descriptor sets were trialled and compared. Examples include constitutional or topological descriptors).
Data preprocessing:
  3. Analysis of the data to ensure its suitability; application of processes such as feature selection, normalization, standardization, and outlier detection. (Specifically in this study, the preprocessing included standardization, neutralization, removing salts, and cleaning the structure, which were processes applied in the OCHEM software, prior to model predictions).
Model development:
  4. Training and validation of the model; development of various machine learning models to obtain high prediction metrics.
Model application and interpretation
  5. Recognition of the applicability domain.
  6. Statistical evaluation and interpretation of the model.

In addition to using examples from literature,^42,43^ care was made to ensure the development followed the principles outlined by the Organisation for Economic Co-operation and Development (OECD). These are guidelines that were formulated in 2002 by QSPR experts to regulate the use and development of these models.^44^ Further details are given in Supporting Information Table S1.

The stages involved in QSPR development are described in the following sections.

### 2.1 Database Development

The data collection method took the form of a data-mining process, often used in model building, where data are gathered by sifting through large amounts of data from the literature in order to obtain new patterns, correlations, or structures in data.^45^ Literature ranging from around 20 years ago to the present was studied to build up two separate databases, and eventually, an overall combined database, consisting of the properties of interest (last cycle sorbent capacity and catalyst methane conversion) as well as the experimental conditions used to obtain these results.

**Figure 2:**
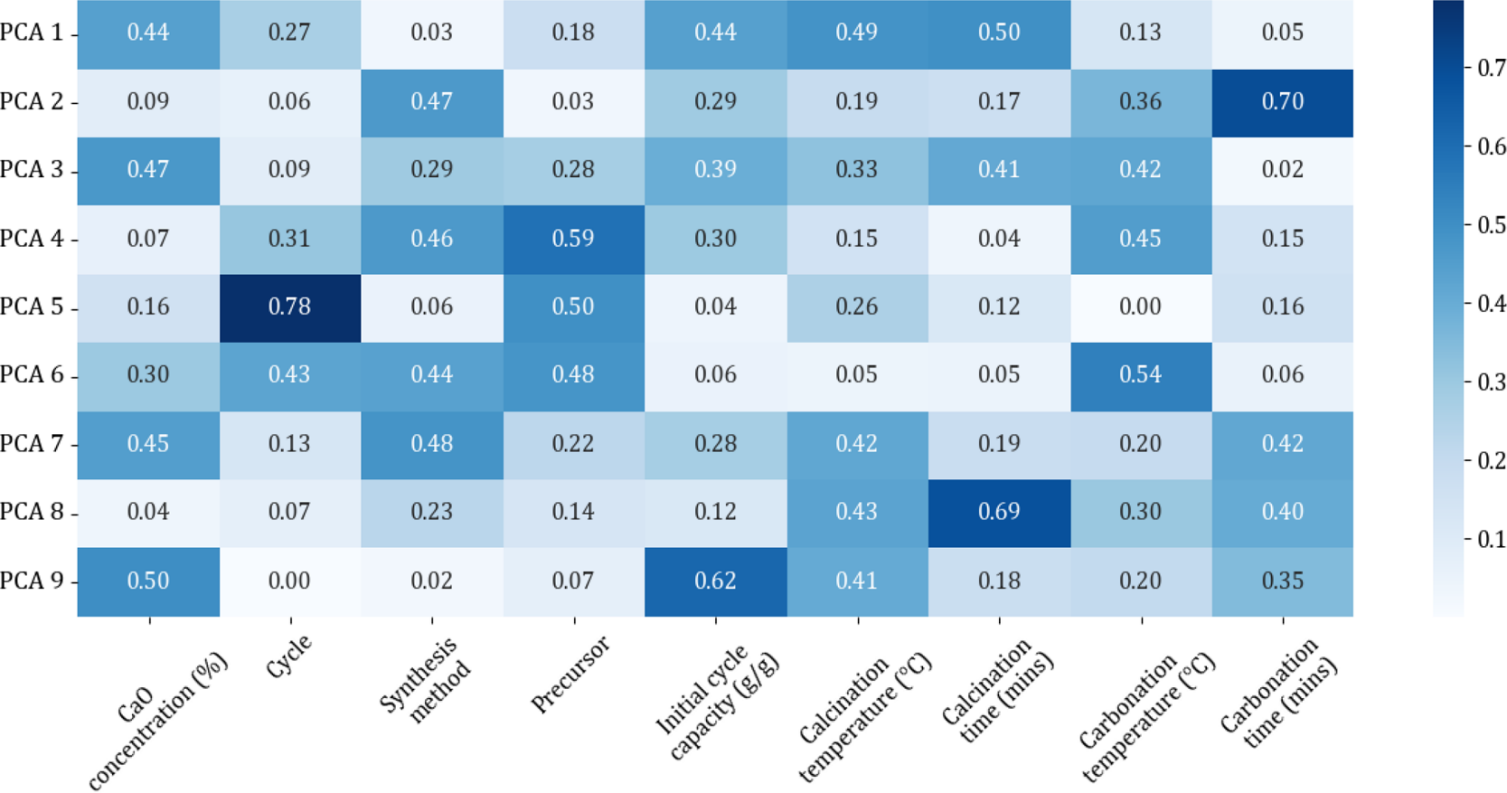
Correlation heatmap of sorbent input features.

**Figure 3:**
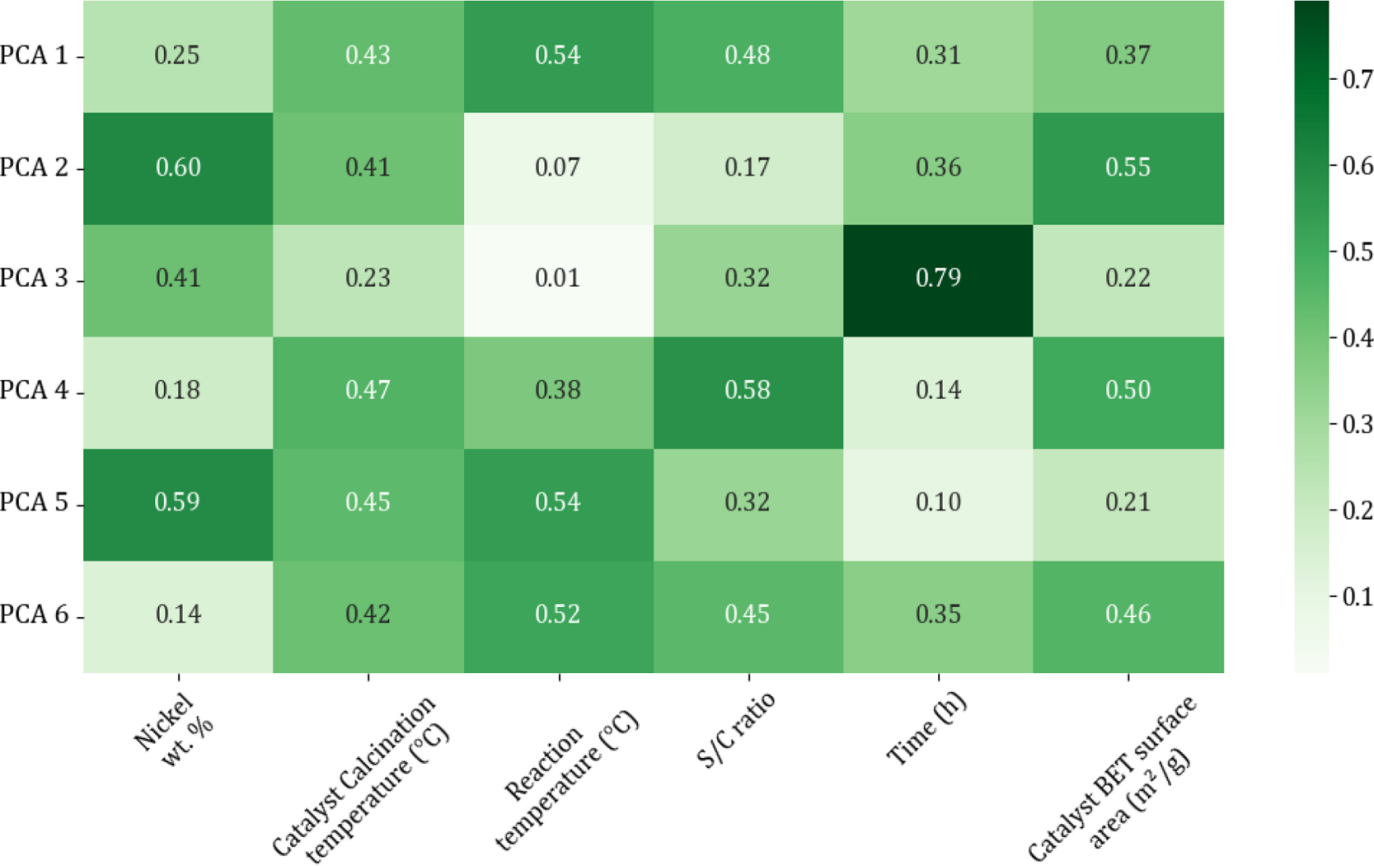
Correlation heatmap of catalyst input features.

Care was taken to ensure the data collected was based on experimental measured data as opposed to computational modeling work. Additionally, some literature was omitted where the properties were present in the work, however with a large percentage of the details of the conditions missing, as this would cause a skew in the ability to predict the properties. For instance, the sorbent database went from a size of 248, to 239, and the catalyst database was reduced from 249 data points to 183, due to the aforementioned reasoning. The conditions chosen to aide in the predictions of the properties are shown in Table 1. These were chosen because they have been proven to have an influence on the properties’ outcome. Additionally, principal component analysis (PCA) was conducted to confirm that all input categories were not redundant (Figure 2 and Figure 3).

**Table 1:** Properties and Their Respective Experimental Conditions per Database

database	property (units)	experimental conditions (units)
sorbent	last cycle capacity (g_CO_2__/g_sorbent_)	CaO concentration (%), cycle number, calcination and carbonation temperatures (°C) and times/(mins), synthesis method, CaO precursor, initial cycle capacity (g_CO_2__/g_sorbent_)
catalyst	methane conversion (%)	nickel concentration (wt %), calcination temperature (°C), calcination duration (h), SMR reaction temperature (°C) fresh BET surface area (m^2^/g), steam/carbon ratio

PCA is a mathematical model that is used to reduce the dimensionality of a data set, while retaining most of the variation in the data set and as an effective procedure for the determination of input parameters.^46^ The reduction of features is achieved by identifying the directions (principal components) where the variation is highest.^47,48^ From the PCA heatmaps the variance across the 9 and 6 principal components (PCs) for the sorbent and catalyst databases, respectively, was shown to be relatively evenly distributed across the first few PCs (e.g., for the catalyst database, the first PC explains 32% of the variance, PC2, PC3, PC4, PC5, and PC6 explain 20.3%, 15.7%, 13.3% 11.6%, and 7.1%, respectively), therefore it was not essentially necessary to remove any parameters. That would be necessary if the first two components contributed to around 70–95% of the total variance (see Table S2 for sorbent variance data).

An additional data set garnered from the PCA heatmaps was feature importance. From Figure 2 it can be calculated that the top three features that contribute to the PCs of the last cycle capacity, are the precursor type, CaO concentration, and calcination temperature, and from Figure 3, the top three to contribute to methane conversion are calcination temperature, S/C ratio, and BET surface area (see Table S3 for full feature importance data).

See Tables S4 and S5 for the individual sorbent and catalyst databases, respectively, and Table S9 for the CSCM literature data.

### 2.2 Molecular Descriptors

Once the experimental conditions were confirmed, the molecular descriptors were evaluated. Molecular descriptors are defined as mathematical representations of a molecule, from characteristics related to the structure of the chemical.^43^ They are calculated from quantum chemistry methods and allow for the modeling of many different properties in fields such as physical chemistry, pharmaceuticals, and analytical chemistry.^49^

The use of different descriptor sets has been shown to result in variable performance in the modeling of the properties of interest.^50^ Therefore, a selection of descriptors were trialled by measuring the accuracy metrics with varying descriptors using the OCHEM platform,^51^ which is an online chemical database with a QSPR/AR modeling environment. A list of the descriptors trialled is given in Table 2. Alongside the molecular descriptors and experimental conditions from the literature, a fundamental input was the structures of the molecules in SMILES format (simplified molecular-input line-entry system), that is, CaO would be represented as O = [Ca].

**Table 2:** Descriptors Used for Comparison^50,52−55^

descriptor	description
ALogPS, OEstate	prediction of logP by ALogPS2.1 program, calculated from each atoms’ intrinsic electronic properties and the influence of other atoms in the molecule
Fragmentor	molecular fragments which contain from 2 to 4 atoms generated by the ISIDA module in OCHEM
GSFrag	descriptors based on the occurrence of certain special fragments
alvaDesc v.2.0.2 (3D)	calculates and analyzes molecular descriptors and fingerprints, such as constitutional, topological, and geometrical descriptors
QNPR	(quantitative name property relationship) uses substrings of SMILES or IUPAC name as a representation of molecules

### 2.3 Machine Learning Models

**Figure 4:**
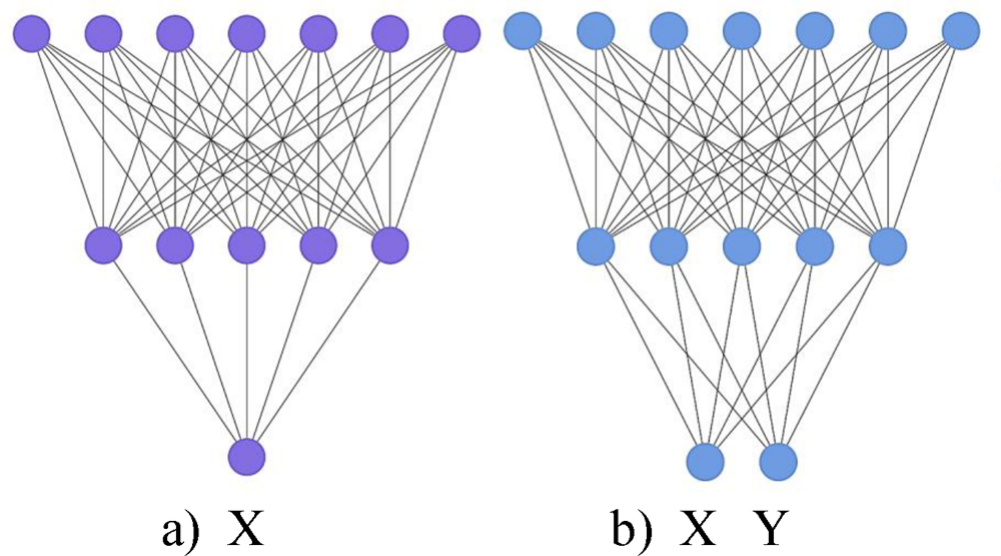
Schematic of a neural network configuration using different inductive transfer approaches: (a) conventional single task learning (STL) and (b) multitask learning (MTL).

Several types of models were used in this study along with the application of the inductive transfer approach. This is the use of a combination of several conventional single task learning (STL) machine learning models, used in parallel, also known as multitask learning (MTL), to act as additional nodes of the original model as opposed to separate models, (shown in Figure 4(56,57)).

### 2.4 Single Task Learning (STL)

The conventional approach of STL in QSPR is to focus on obtaining the target of one property, using only descriptors and conditions relating to that single property. This was the first approach taken to predict the properties of last cycle capacity and methane conversion using their respective databases, alongside comparing performance with descriptors. The machine learning models trialled were associative neural network (ASNN), deep neural network (DNN), and least-squares support-vector machine (LSSVM). These models were chosen as they can be applied as MTL models, as well as with the use of molecular descriptors on the OCHEM platform.

**Figure 5:**
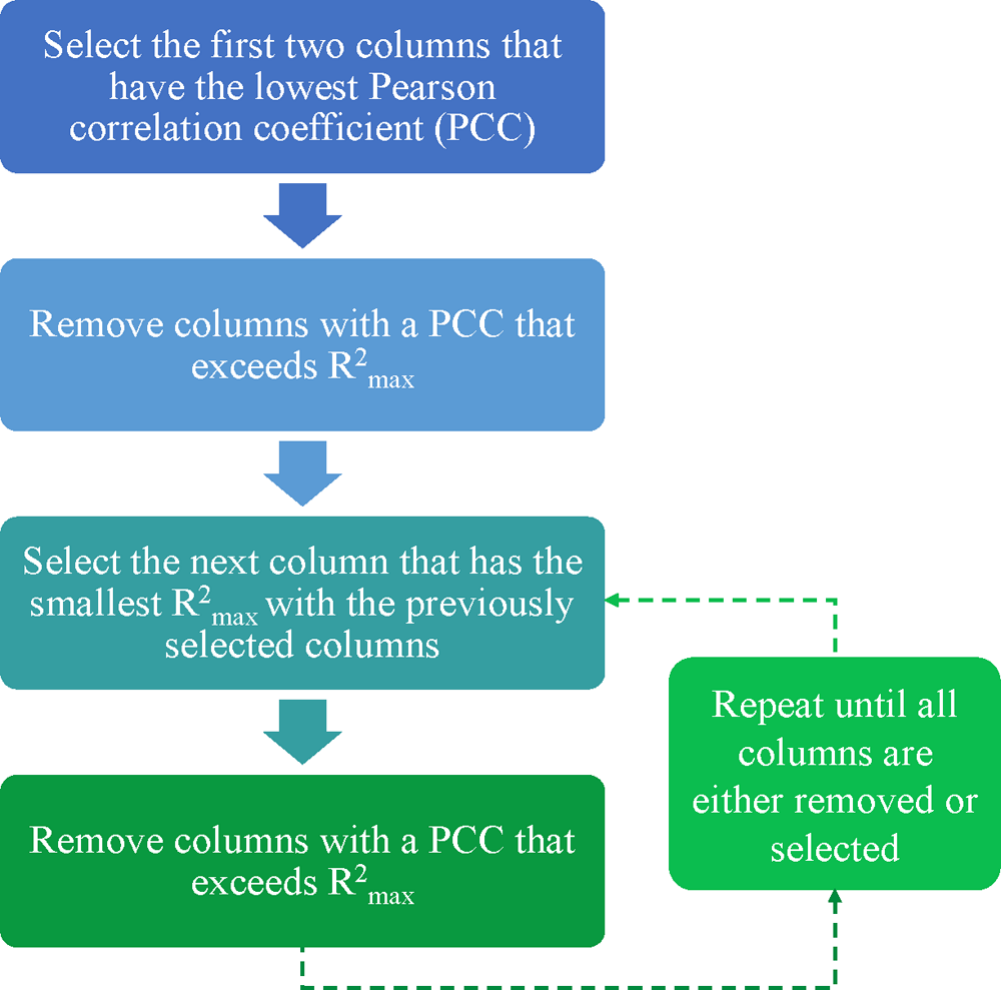
Process flow diagram showing the steps involved in unsupervised forward selection (UFS) to remove unneeded molecular descriptors.

A built-in function of OCHEM called unsupervised forward selection (UFS) was implemented. This is a method used for eliminating redundant descriptors and is specifically used in the development of QSPR models. Redundancy can be common in QSPR data sets due to high or exact linear dependencies between subsets of the variables, and high multiple correlations between subsets of the variables. These factors hinder the development of models that have the ability to effectively predict new data. UFS produces a reduced data set that contains no redundancy and has a minimal amount of multicollinearity, via the method depicted in Figure 5(58) (see Supporting Information for other descriptor filter settings in addition to UFS).

ASNN, DNN, and LSSVM were implemented in OCHEM, and then compared against each other to first observe the effect on ability to predict the properties using molecular descriptors with experimental conditions, then the best performing algorithms were used to further predict unseen properties using more data from the literature. Details on the architectures of the machine learning models can be found in the Tables S6–S8.

### 2.5 Multitask Learning (MTL)

With MTL, multiple properties are trained and learned in parallel.^59^ This approach improves the performance of STL due to the training data from the extra tasks acting as a suggestive bias, adding in effect constraints for the other task at hand, which aids in the accuracy and the speed of learning.^60^

Two types of comparisons were conducted, the first being a combination of finding the machine learning model and molecular descriptor set, which resulted in the highest prediction accuracy. For efficiency, this was trialled using STL data only, and the models were conducted using OCHEM’s automatic multiple models setting. The results from this were used in the second comparison which was STL compared against MTL, all using default model settings, to observe which inductive transfer approach was the best performing for this data.

Following this, the approach that yielded the best accuracy metrics was then taken and the model settings were optimized to obtain the best predicting model, which was then used to predict unseen CSCM molecules.

### 2.6 Model Development and Performance Measurement

Each model was validated using a grid search method based on 5-fold cross-validation procedure, which is useful to mitigate overfitting, parameter optimization as well as evaluating the predictive validity of linear regression models.^61^ On the OCHEM platform, the five-cross validation process develops a new model on each validation step without the use of known information about the molecule, as they are only calculated following the completion of the model developed. This approach is reported to be the correct validation approach, as no prior information about the test molecules is used to skew the models’ development.^50^ Additionally, the models themselves had internal validation which can be seen in the Supporting Information.

To assess the predictive performance, the accuracy metrics used were root-mean-square error (RMSE), mean absolute error (MAE), and the coefficient of determination (*R*^2^), shown in eqs 4 to 6, The aim of the machine learning models was to obtain the highest *R*^2^ and lowest RMSE and MAE, where *n* is the quantity of data required for the network training,  is the predicted value of *y*, *y*_*i*_ is the target output, and  is the mean value of *y*.
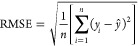
4
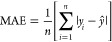
5
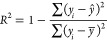
6Additionally, as a recommended tool when dealing with large data sets, in order to evaluate the models’ prediction capabilities of new molecules, the absolute relative error (ARE) for each molecule was calculated using eq 7 and the models’ average (AARE) was calculated, eq 8, where *n* is the number of molecules predicted per model, and *i* is the predicted molecule.^62^
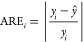
7
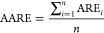
8

## 3 Results and Discussion

### 3.1 Comparison of the Models

**Figure 6:**
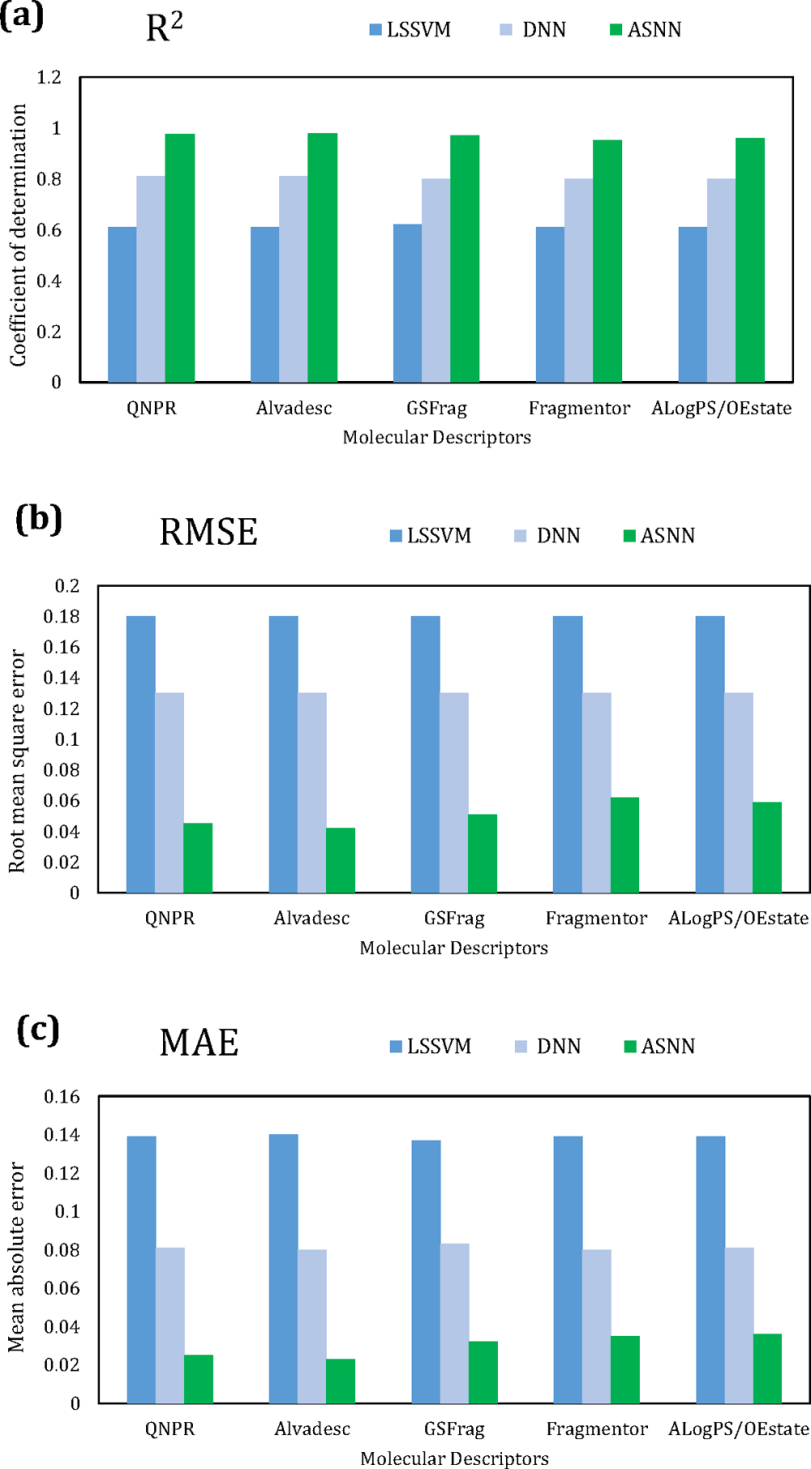
Comparison of machine learning models—least-squares support vector machine (LSSVM), deep neural network (DNN), associative neural network (ASNN), and molecular descriptors on the catalyst methane conversion database: (a) coefficient of determination (*R*^2^), (b) root-mean-square error (RMSE), and (c) mean absolute error (MAE).

A comparison of molecular descriptors and machine learning models for the sorbent database, Figure 6, shows that the ASNN model gave the highest *R*^2^ and lowest RMSE, across all models. In terms of the “most optimal” set of descriptors, although not clear in the figure, AlogPS/OEstate, Fragmentor, and GSFrag resulted in equally strong values, while QNPR and alvaDesc consistently underperformed for this database. On the other hand, interestingly, as shown in Figure 6, in a comparison of machine learning models and descriptors for the catalyst database, the best performing model was in agreement with the sorbent database (ASNN), whereas the descriptors that performed best were the opposite. Here, alvaDesc and QNPR were the top two and Fragmentor was the least accurate predictor.

ASNN stood out with both the catalyst and sorbent results, and suggestions for this include the learning method behind this algorithm. It is a combination of an ensemble of the feed forward artificial neural network model (ANN) and the K-nearest neighbor technique (kNN). ANN is a supervised learning algorithm, which does not require any preassumption of the input–output relationship.^63^ This would have a positive effect on the prediction capability as it would allow the model to rely more on the molecular descriptors calculated to provide a prediction. The model uses the correlation between ensemble responses as a measure of distance within the analyzed cases for the kNN,^57^ which corrects the bias of the neural network ensemble.^64^ The combination of two algorithms, proved to be optimal in this case.

As all five descriptors resulted in high predictions for either of the databases, it was too ambiguous to decide which was optimal at this stage, therefore it was decided to take all sets to the next step of applying the inductive transfer approach of multitask learning, to see if what effect, if any, this approach had on the results, which yielded interesting results.

**Figure 7:**
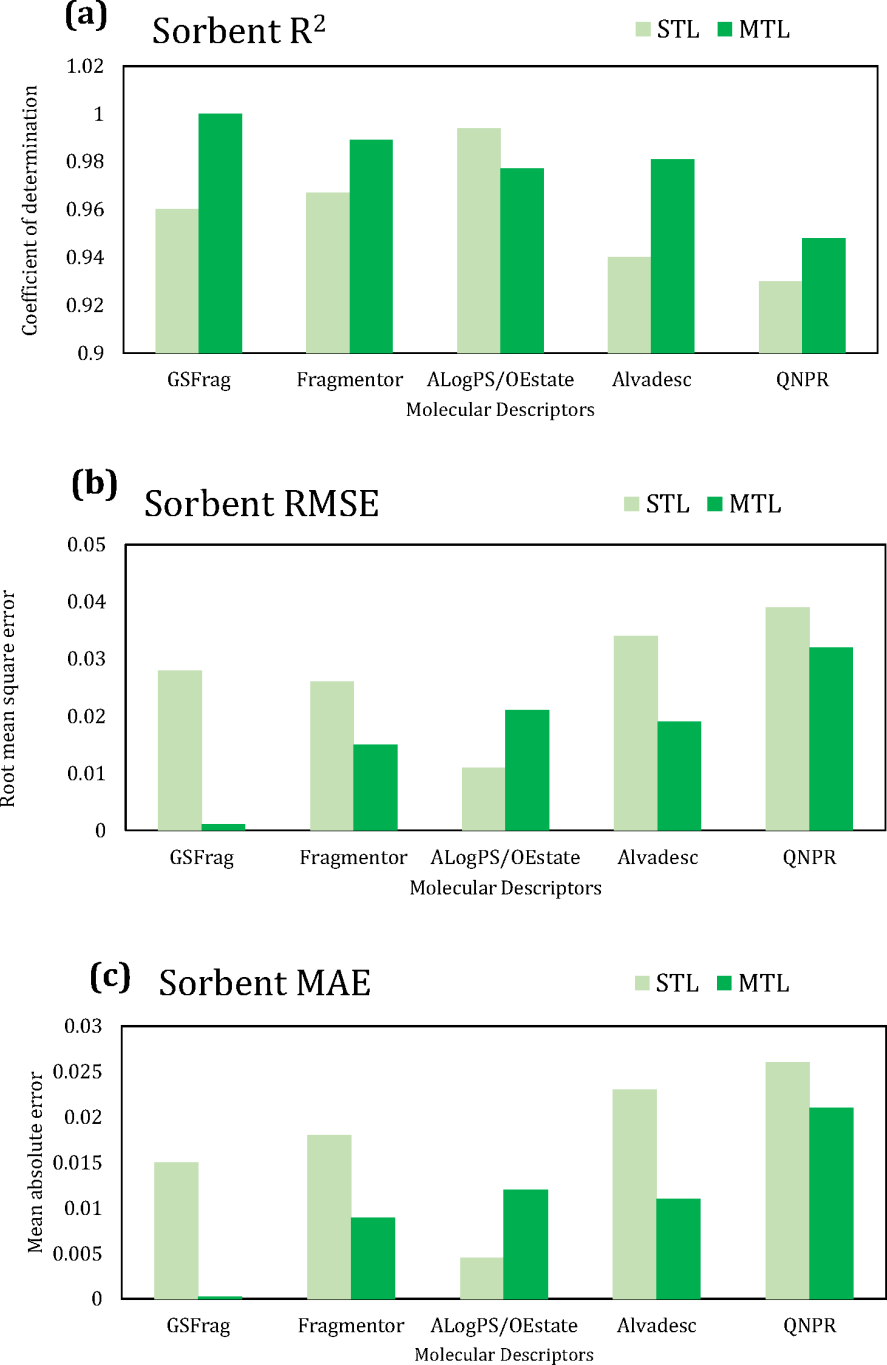
Comparison of the effect of the inductive transfer approach and molecular descriptors, on the associative neural network (ASNN) algorithm, for the sorbent database: (a) coefficient of determination (*R*^2^), (b) root-mean-square error (RMSE), (c) mean absolute error (MAE).

**Figure 8:**
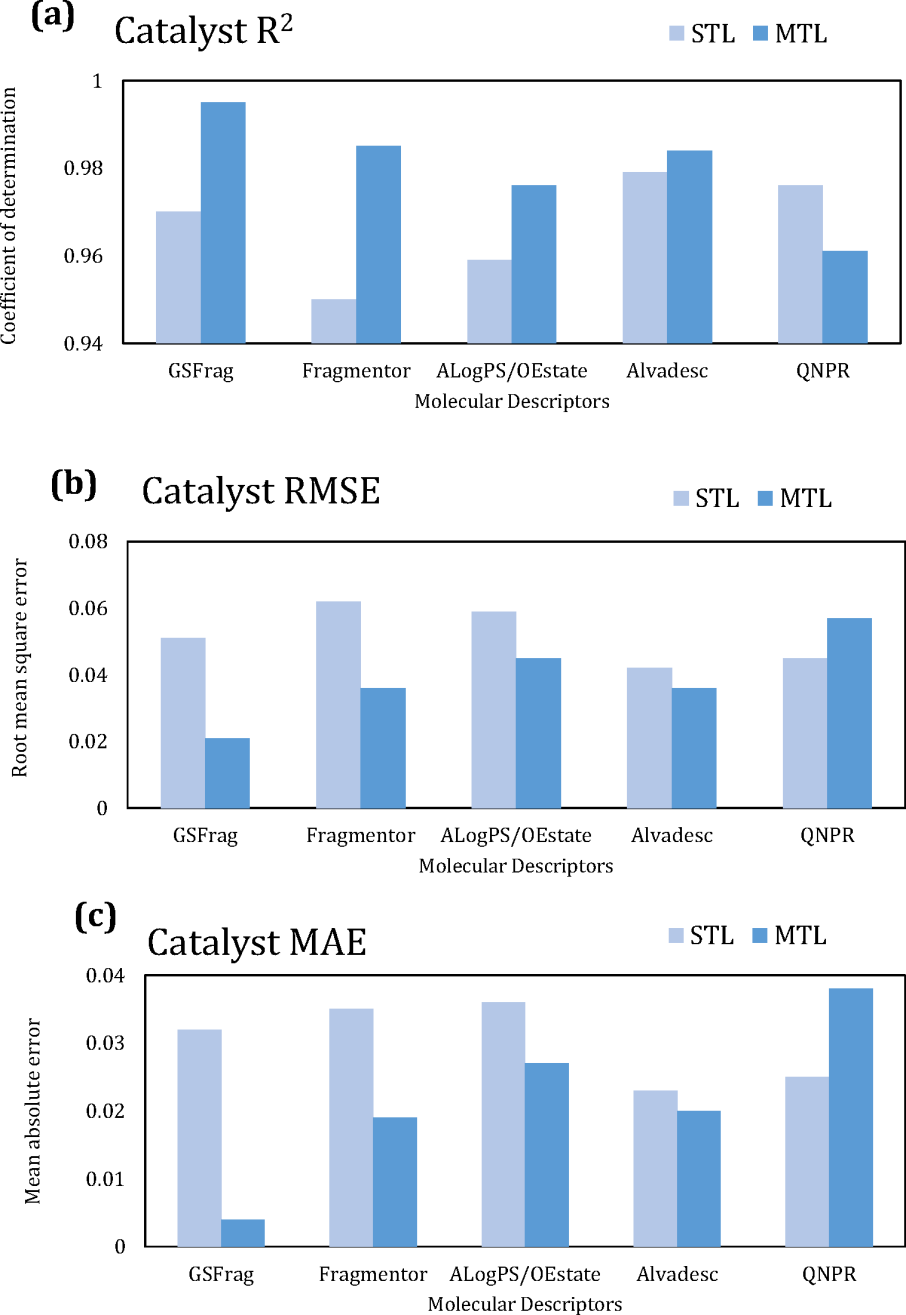
Comparison of the effect of the inductive transfer approach and molecular descriptors, on the associative neural network (ASNN) algorithm, on the catalyst methane conversion database: (a) coefficient of determination (*R*^2^), (b) root-mean-square error (RMSE), and (c) mean absolute error (MAE).

Figure 7 gives the accuracy metrics for sorbent database, and Figure 8 gives the results for the catalyst database, using the ASNN model. The results show that MTL gave the highest *R*^2^, and the lowest MAE and RMSE. The MTL improved accuracy predictions over STL mainly due to having additional data points, with 422 for the MTL compared to 239 and 183 for the sorbent and catalyst STL models, respectively. Extra data are not always a good thing and, instead, the quality of data has more bearing, due to the effect of overfitting. If the model is tuned with too much data, it starts to memorize data instead of “learning”, which leads to high errors for unseen data.^65^ However, it has also been shown that more data can lead to lower estimation variance and therefore better predictive performance. More data increase the probability that they contain useful information, which is advantageous.^66^ With these opposing arguments in mind, for this particular data set, it is suggested that the additional data points improved the generalization of the prediction, by using the domain-specific information contained in the training signals of the added related property, which allowed for these training signals to act as an inductive bias.^56^

As mentioned before, the molecular descriptors gave interesting results between the two databases, with AlogPS/OEstate and Fragmentor descriptors performing well with the sorbent database, and alvaDesc and QNPR giving good results for the catalyst database. As seen in Table 2, AlogPS/OEstate calculates descriptors based on each atom’s intrinsic electronic properties, as well as looking at the counts of atom or bond types and Fragmentor set of descriptors focus on molecular fragments which contain between 2 and 4 atoms. The QNPR set of descriptors uses substrings of SMILES as a representation of molecules,^50^ and alvaDesc calculates descriptors such as constitutional indices, charge descriptors, and atom pairs for example, with most descriptors being 2D based. When looking at the two databases, a main difference is first the number of experimental conditions as inputs; however, this is unlikely to affect the way the descriptors behaved with the databases, and another difference is the SMILES format. As a percentage, more of the sorbent database had a SMILES format that incorporated bonds and functional groups (31% compared to catalyst 22%), whereas the catalyst database had more data that was a “mixture”. This could explain why it yielded good results for the catalyst data set. Typical molecules used in QSPR are standard compounds and therefore the SMILES input often involves structures such as aromatic rings, functional groups, and the bond type, for example.

As most of the catalyst molecules included in this study are not whole compounds but rather a physical mixture, the SMILES input format, in this case, was simply the element in the molecule along with its charge, as a string. Therefore, considering that QNPR relies on the substrings to calculate descriptors as opposed to focusing on bonds or functional groups, it is suggested that it could readily calculate more accurate and relevant descriptors to the databases used in this study. Additionally, alvaDesc looks at aspects such as charge descriptors, and 2D matrix-based descriptors for example which would favor the catalyst database based on the SMILES input. Conversely, looking at the sorbent database, the reasoning behind why AlogPS/OEstate and Fragmentor would be favorable here, is that it specifically looks at the bond types, as well as the molecular fragments and neighboring atoms, which would allow for better prediction if the SMILES input data consisted of this information.

In terms of STL vs MTL, as seen in Figure 7 and Figure 8, the descriptors that favor the STL approach for the sorbent database, that is, the set that yields higher *R*^2^, and lower RMSE/MAE, in order is AlogPS/OEstate > Fragmentor > GSFrag > alvaDesc > QNPR. For the STL catalyst database the performance is alvaDesc > QNPR > GSFrag> AlogPS/OEstate > Fragmentor, which is in line with the aforementioned reasoning. Interestingly, the set of molecular descriptors that sit in the middle for both databases in terms of predictive ability, is GSFrag. For this reason, it is the best performing set of descriptors for both databases when used with the MTL approach, as it provides equally strong performance for each database, thus resulting in an overall high performing prediction capability when a combined sorbent and catalyst database is the input data.

The GSFrag descriptors are the occurrence numbers of certain special fragments containing 2–10 non-hydrogen atoms, and it is been proven that the occurrence of specific fragments produces a unique code of a chemical structure for wide sets of compounds.^67^ As most QSPR descriptors sets are predominately developed on the field of drug discovery, it is unlikely that other descriptors (such as Fragmentor, which is based on the pharma industry), to have fragments common to sorbents and nickel catalysts. QSPR equations constructed from these descriptors usually provide good statistical characteristics and high predictive ability. Molecular fragments of this type provide good correlations between properties and chemical structure for many classes of compounds.^68^

Consequently, as the results indicated that the GSFrag was favorable, along with the MTL ASNN approach, these settings were taken forward to predict new unseen CSCM molecules.

When predicting new unseen molecules using this ML model setup, although the accuracy metrics were almost perfect, the prediction capability was less than optimal, giving a range of accuracy between 11 and 300% between the actual vs predicted values for both the methane conversion and last cycle capacity. This can be due to a number of reasons, namely overfitting and having the experimental conditions skew the effects of the molecular descriptors.^65^ In this case what may have occurred can be due to either the limits of the data, either from being limited in size or having too much “noise”, or it may be down to the constraints of the algorithm itself, which may be too complicated or too simplified to this type of data.^69^ It is difficult to say, but the molecular descriptors may also have a limitation on the prediction abilities as they are seldom used in sorbent and catalyst materials, therefore the ability to “learn” is only as good as the past research in which they will have been used.

Because of these various reasons, in order to reduce the cumulative discrepancies on the end results, the machine learning model development stage was repeated with some omitted steps. First, the only model taken forward was the ASNN as that outperformed the other two clearly. Additionally, GSFrag was the molecular descriptor set taken forward because it resulted in equally good results for both databases. What was changed however was the data set inputs, the validation, and the model architecture. After trial and error and using data from the PCA information, a new model that resulted in more accurate predictions was developed, with the input data and ASNN structure given in Table 3.

**Table 3:** Properties of the Final ML Model Used for the Prediction of Unseen Combined Sorbent Catalyst Materials (CSCM)

database	property (units)	experimental conditions (units)
sorbent	last cycle capacity (g_CO_2__/g_sorbent_)	CaO concentration (%), cycle number, calcination and carbonation temperatures and times (°C/mins), synthesis method, CaO precursor, initial cycle capacity (g_CO_2__/g_sorbent_)
catalyst	methane conversion (%)	nickel concentration (wt %), calcination temperature (C), calcination duration (h), SMR reaction temperature (°C) fresh BET surface area (m^2^/g), steam/carbon ratio
validation		5 cross-validation

ASNN architecture	
training method	SuperSAB
number of neurons in hidden layer	32
learning iterations	1000
ensemble	256
additional Parameters	partition = 3, selection = 2

### 3.2 Applicability Domain

The Applicability Domain (AD) is a theoretical region in “physicochemical space” on which the training set of the model has been developed, and for which a QSPR model should make predictions with a given reliability.^70,71^ Due to this, it is recommended that new data be predicted within the AD by interpolation as opposed to extrapolation. In this study the AD was well-defined with validation data falling within the AD; however, the prediction results for the unseen CSCM molecules did not fall within the AD, and therefore they were calculated by extrapolation. To visualize the spread of data a distance-to-model plot (Williams plot) was generated, which assesses how “far” a molecule is from the model. Compounds that are “further from the model”, are expected to have lower prediction accuracy than compounds that are “closer”.^72^

**Figure 9:**
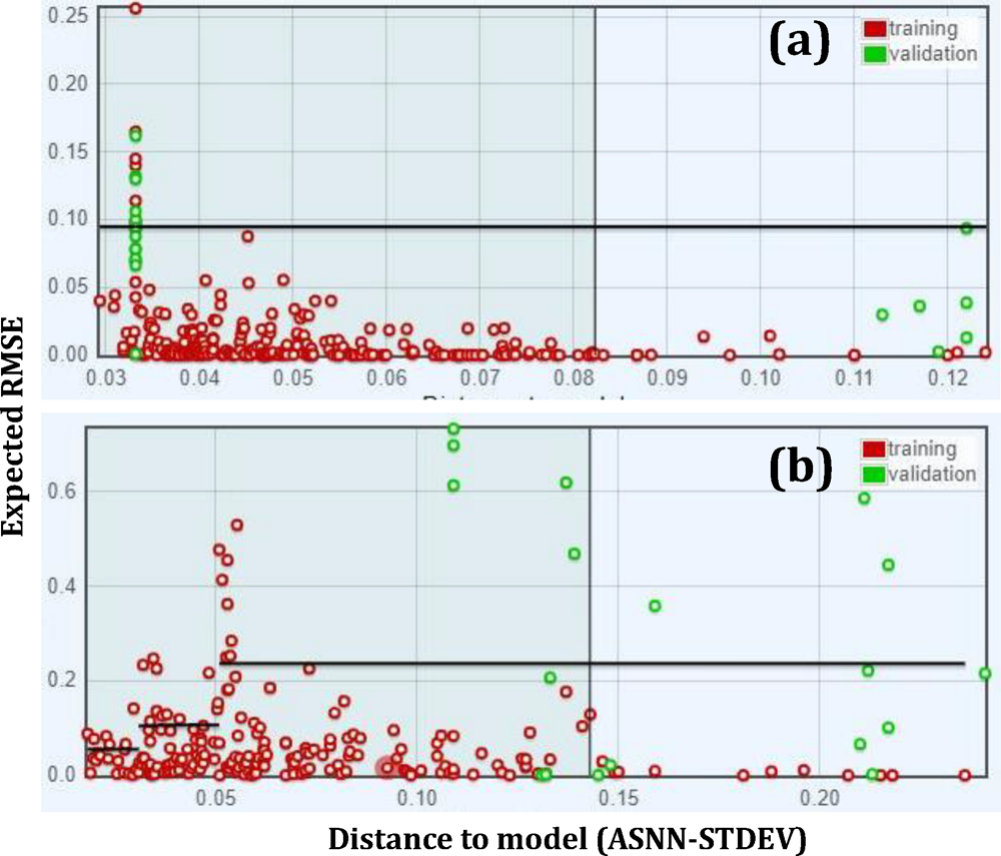
Williams plots for the model when applied to predict unseen CSCM molecules, with ASNN-Standard Deviation used as the distance to the model. Black lines represent the averaged RMSE over different distance-to-model intervals: (a) last cycle CO_2_ capacity (g_CO_2__/g_sorbent_) and (b) methane conversion (%).

Figure 9 gives the Williams plot for the graphical visualization of outliers for the predicted results in the developed model. The red dots indicate the prediction capability, where its *X*-position is the “distance-to-model” given by ASNN-Standard Deviation (STDEV) and its *Y*-position is the expected RMSE. From this, it can be suggested that OECD guideline 3 is adopted, as the predicted data for the model development largely falls within the AD.

Conversely, when applying this model to new unseen data, the results are not as fruitful. The green data points in Figure 9, which are the new predicted compounds, all indicate an RMSE value of, or around, 0.086 and 0.4 for the sorbent and catalyst properties, respectively. This is expected as when looking at the original metrics, the RMSE values for the developed models were 0.09 ± 0.03 for the sorbent training data and 0.23 ± 0.08 for catalyst training data. Therefore, the expected RMSE for the predicted CSCM molecules is in line with the developed model training data, with the catalyst RMSE being slightly higher. In terms of distance-to-model, they are on the high end, meaning the predictions are not as reliable as they could be. Reasons for why this occurred could be due to the nature of the input data vs the CSCM predictions.

However, distance-to-model plots estimate the reliability of predictions, and while accuracy is an objective measure that has a fixed calculation procedure, reliability is subjective and can be estimated in numerous ways.^73^ Because of this, data that falls out of the AD is not necessarily invalid, however, it is less reliable.

### 3.3 Model Prediction Capability

The GSFrag/ASNN model was used to predict the last cycle capacity of CSCM molecules, again where the data was collected through literature data-mining, the difference being that the new molecules possessed both catalytic and sorbent properties, compared to the molecules used to create the predictive model, which had one or the other. As the study of CSCMs is not as exhaustive as that for nickel-based catalysts and calcium-based sorbents, there were less data available to which to apply the model, with only 41 data points. Furthermore, with some of the data collected, some literature did not provide both last cycle capacity and methane conversion despite reporting on a CSCM. The database along with the predicted properties can be found in Tables S9 and S10. Lastly, through trial and error, a pattern was observed in the type of input data fed into the machine learning model. A combined sorbent and catalyst database made up of just the 239 and 183 data points (422 data points) yielded a model that could not predict the output behavior at all, that is, no clear regression pattern was seen in the actual vs predicted graphs. Consequently, splitting the CSCM database of 41 data points into two and adding some to the overall MTL database (resulting in 446 data points overall) and using some as the validation unseen data (17 data points) was successful, and the model was able to “learn” and predict some of the CSCM behaviors.

**Figure 10:**
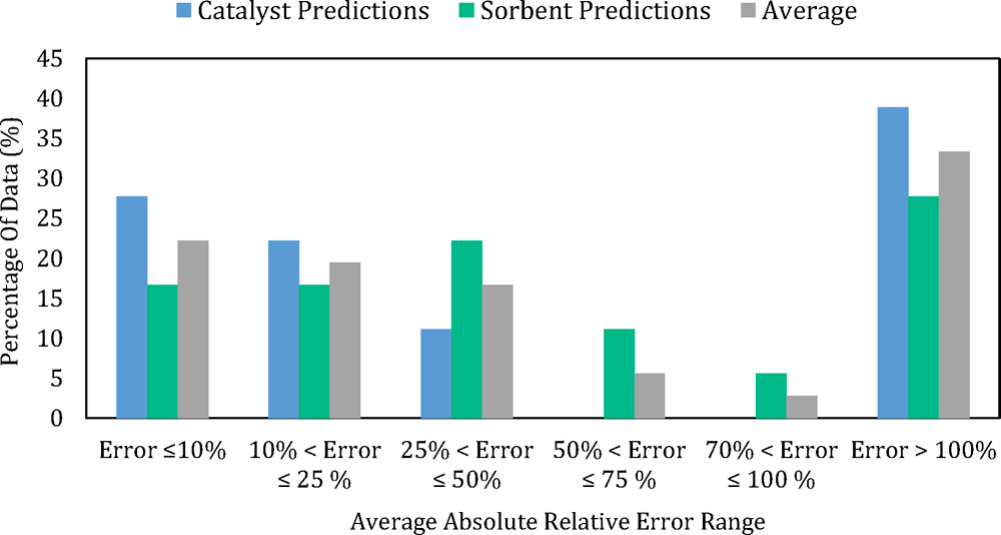
Comparison of the percentage of data belonging to each average absolute relative error (AARE) range for the last cycle capacity and methane conversion prediction of CSCM molecules.

Figure 10. shows the percentage of molecules’ predictions that fell in specific AARE ranges (%), for the last cycle capacity prediction, the methane conversion prediction, and an average of the two for the unseen CSCM molecules. From this figure, it is clear that although a meaningful amount of unseen CSCM were predicted well (around 30–35% of data falling in an error range of 10% or less), a significant amount also fell in the error range above 100%.

The molecules with the highest ARE (%) were those at the extremities, in this case, the molecules with very low sorbent capacities, the molecules missing highly influential conditions (e.g., CaO concentration %), and molecules with conditions that were not commonly occurring in the databases that developed the model. In these cases, the information provided was not sufficient for the model to extrapolate well, based on existing data.

The average prediction capability of the overall model is not tremendously high, with only 58% of the data resulting in an AARE of 50% or less; however, given this new approach, and the fact that high ARE (%) values have an explanation behind them, the models’ application on new data is a fairly positive one.

Looking at the prediction capability of the individual properties, the catalyst properties are predicted ever so slightly better at 61% of data falling in an AARE range of 50% or less, compared to 58% for the sorbent properties. This occurs because the catalyst database uses fewer experimental conditions and relies on the molecule more to predict the behavior. This can be a good and bad thing; it allows for less literature data to be gathered to predict an output from ML and QSPR. On the other hand, it prevents the ability to observe correlations in the input data, which can help further the development of a CSCM, as described in the next section.

### 3.4 Patterns in Data

**Figure 11:**
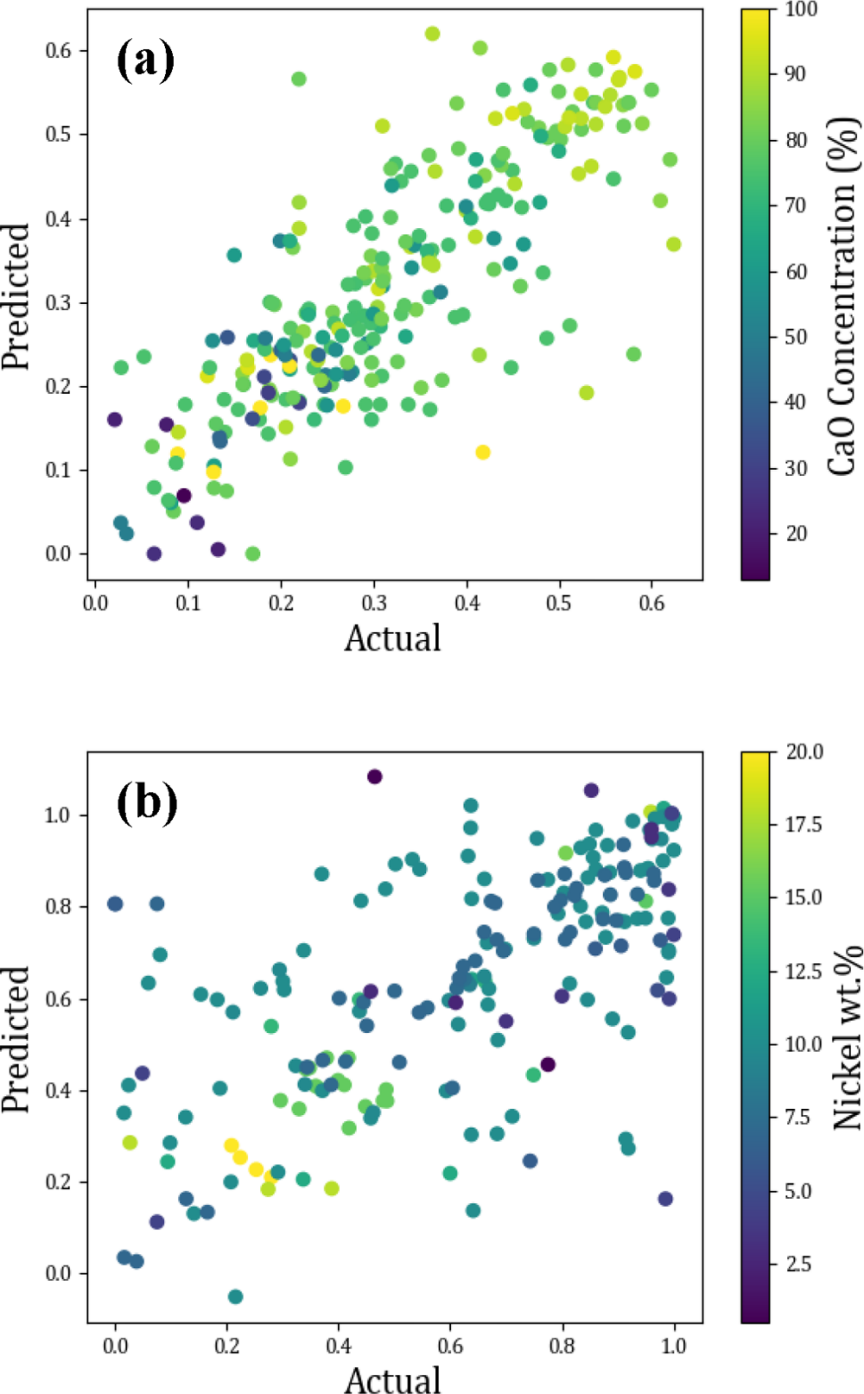
Actual vs predicted scatter plots showing the effect of an influential input for (a) last cycle capacity (g_CO_2__/g_sorbent_) as a function of CaO concentration (%) and (b) methane conversion (%) as a function of nickel wt %.

Further to obtaining a measure of how well the model could predict new molecules, additional information was also garnered from the results. Patterns in the output prediction data were seen as a function of input data, specifically the two main influencers CaO concentration (%) and nickel wt % (Figure 11).

Figure 11 (a) and (b) show certain patterns in the input data; a low concentration of calcium oxide in a sorbent material yields lower CO_2_ capacities in the last cycle, as expected, but there is also an optimal concentration, as too much CaO % also does not yield the maximum CO_2_ capacity in the last cycle. Similarly, the wt % of nickel in a catalyst is shown to yield low conversion if it is too high. In both cases, this is likely to be due to dispersion and availability of active sites.

**Figure 12:**
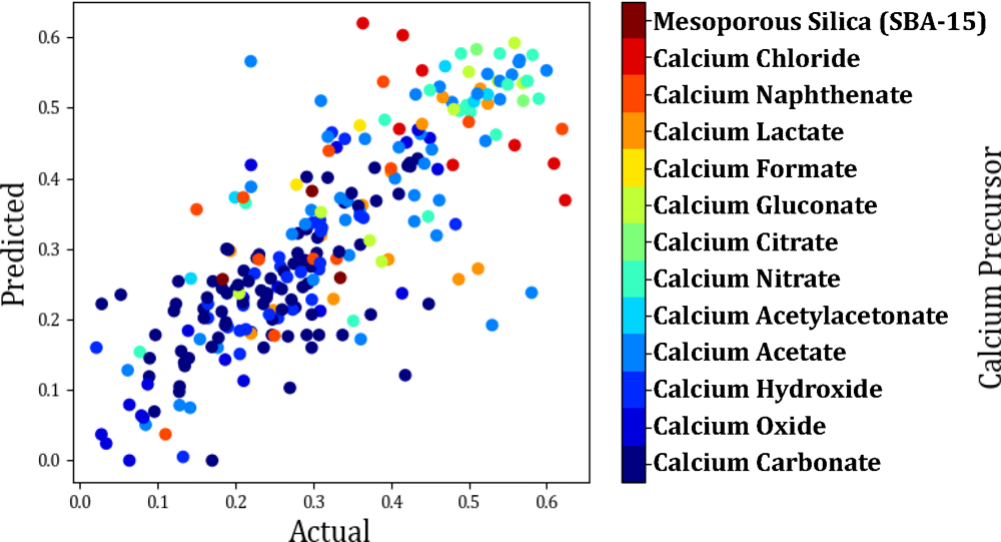
Actual vs predicted scatter plot showing the effect of the calcium precursor on the last cycle capacity (g_CO_2__/g_sorbent_).

Additional parameters have an effect on where a data point sits on an actual vs prediction plot, therefore color plots such as these, prove helpful to narrow down the best direction to go in CSCM development. Further information garnered from color plots generated from this study include the calcium precursor and the inert support in the sorbent. As you can see from Figure 12, there is an indication that the naturally occurring precursors of calcium carbonate and calcium oxide, yield a lower last cycle capacity. Conversely, the best performing precursors are shown to be calcium gluconate and calcium nitrate. Other precursors that gave good performance but equally underperformed were calcium acetate and calcium chloride. The reason for this is that it is well-known that calcium oxide from natural precursors undergoes a rapid loss of reactivity after several carbonation/calcination cycles. Additionally, it has been reported that natural sorbents, such as limestones, form product layers with higher cohesion than those formed by the synthetic sorbent. Thus, once the small pores have largely filled and a thin layer of product has been deposited, the natural sorbents CO_2_ uptake rapidly decreases.^74^

**Figure 13:**
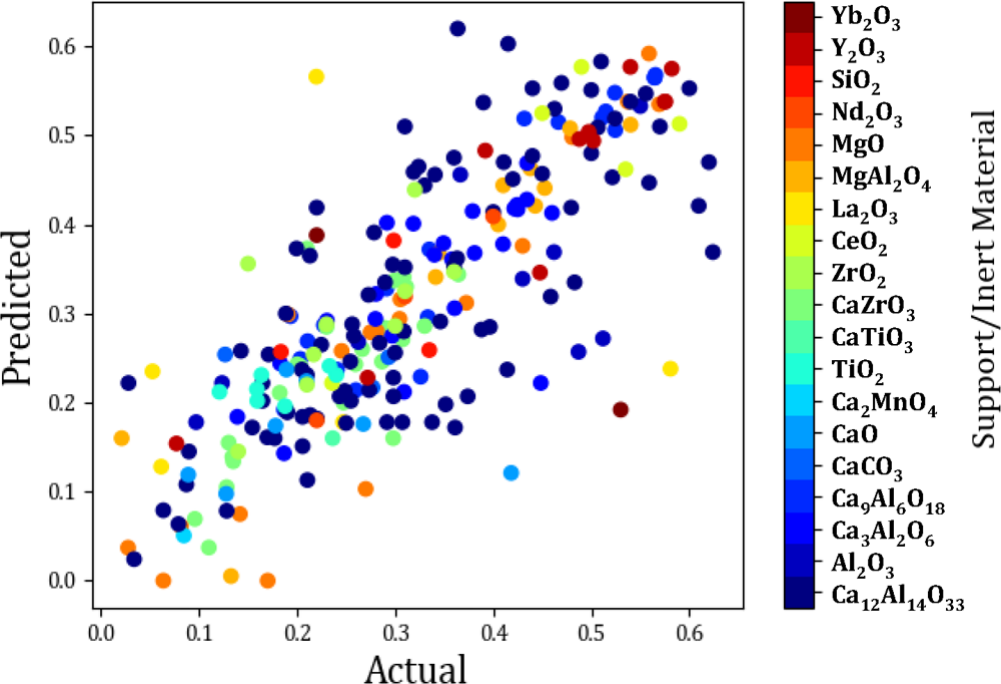
Actual vs predicted scatter plot showing the effect of the inert support on the last cycle capacity (g_CO_2__/g_sorbent_).

**Figure 14:**
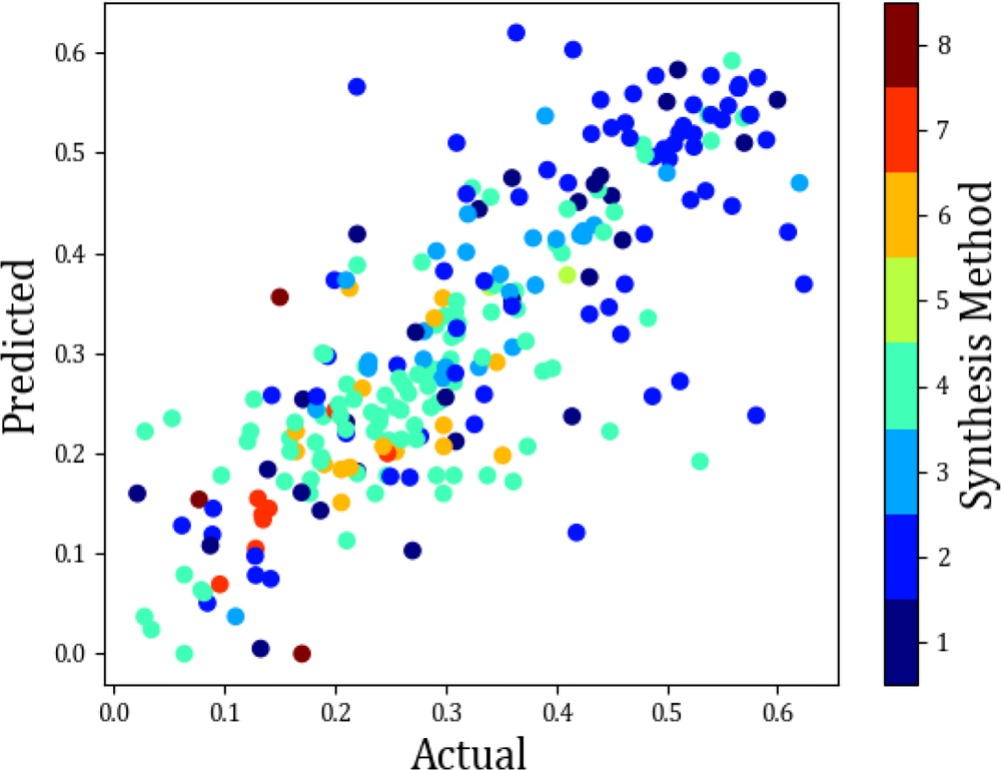
Actual vs predicted scatter plot showing the effect of the synthesis method on the last cycle capacity (g_CO_2__/g_sorbent_)

From Figure 13 it can be seen that Mayenite (Ca_12_A_l1_4O_33_), provides both good and underpar performance. From this color plot, very little information can be gathered, in terms of the type of support that is optimal for the sorbent development. This can be due to the fact that there are other more influential factors, and that the inert support, as inferred from the name, has little effect on the overall performance. A color plot that did provide a definitive output is the synthesis method, Figure 14 and Table 4. From this color plot, it can clearly be seen that the sol–gel method outperforms the other methods consistently. A method that consistently underperformed was the surfactant template method/ultrasound-assisted technique. Reasons for this include that the former is a well-researched method and has had time to be perfected in time, whereas the latter is a fairly new and novel method.

**Table 4:** Key for the Synthesis Method

	synthesis method
method 1	mixing
method 2	sol–gel/sol gel combustion/sol mixing/gel template
method 3	flame spray pyrolysis (FSP)/flame synthesis/combustion synthesis
method 4	wet mixing/mixing + pelletization
method 5	four-step heating method
method 6	coprecipitation
method 7	surfactant template/ultrasound-assisted technique
method 8	wet impregnation

Additional information gathered from other color plots for the sorbent properties (see Figures S4 to S6) was data such as the optimal cycle number, calcination/carbonation time, and calcination/carbonation temperature.

**Figure 15:**
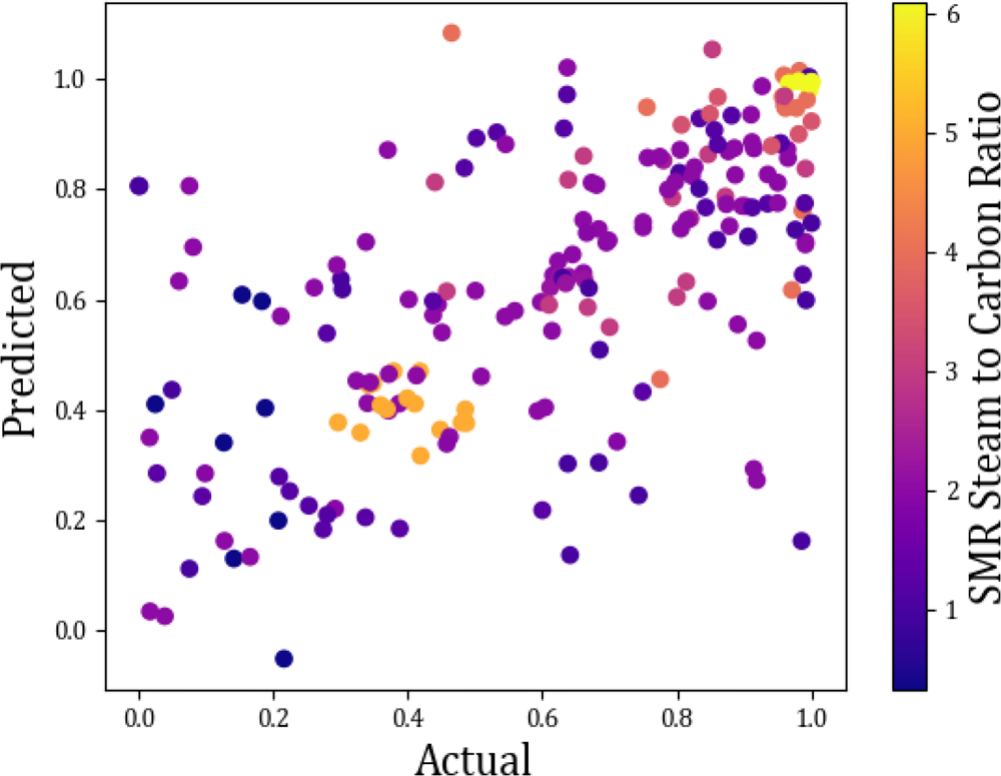
Actual vs predicted scatter plots showing the effect of steam to carbon ratio on methane conversion (%).

**Figure 16:**
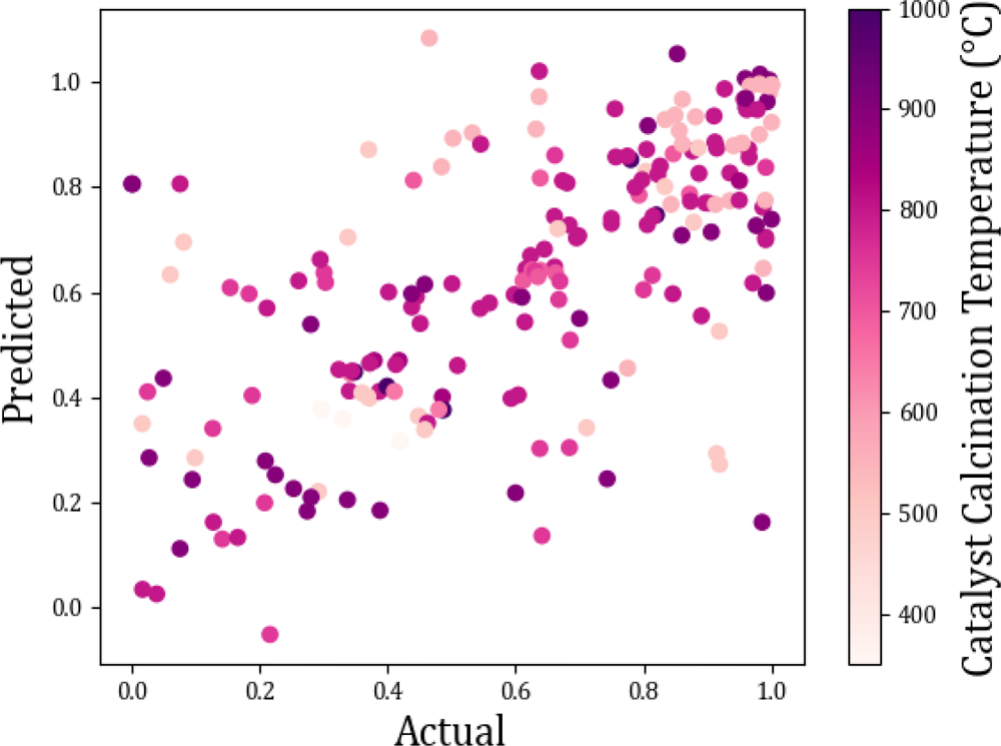
Actual vs predicted scatter plots showing the effect of catalyst calcination temperature (°C) on methane conversion (%).

**Figure 17:**
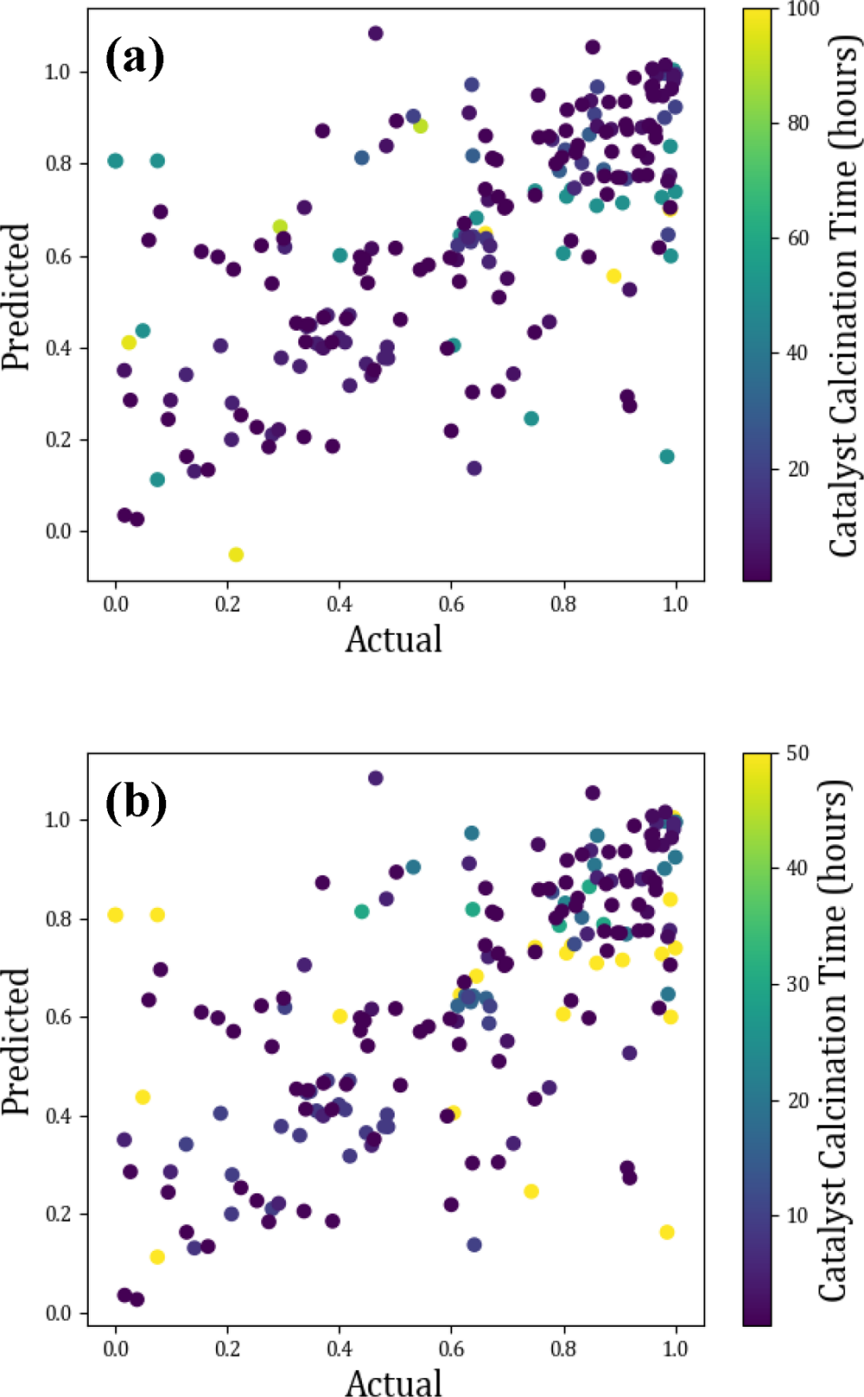
Actual vs predicted scatter plots showing the effect of catalyst calcination time (a) maximum 100 h, (b) 50 h on methane conversion (%).

Data gathered from the catalyst color plots equally gave some conclusive and some ambiguous results. Figure 15 represents the optimal steam-to-carbon ratio during the reforming step. Clearly its shown that the higher is the S/C the higher is the methane conversion (%). Likewise, from Figure 16, it can be seen that an average calcination temperature for the catalyst, at around 600 °C, results in a high methane conversion (%). On the other hand, a color plot that seemed inconclusive is Figure 17, the catalyst calcination time. Even with changing the *y*-axis to a smaller time range, the data did not provide a definitive time, as shown in both color plots. What can be inferred from this plot is that a relatively low time, for example, 10 h, is sufficient.

Additional information gathered from other color plots include sorbent properties such as cycle number, carbonation/calcination time and temperature, and catalyst properties such as BET surface area and the SMR reformer temperature (see Figures S1–S4.) When all the results from the actual vs predicted plots are collated, the results of the route that should be explored to develop a CSCM that has good performance with respect to both the last cycle capacity and the methane conversion are shown in Table 5.

**Table 5:** Data Gathered from the Color Plots, Indicating Parameters That Lead to a Good/Poor Performing CSCM

parameter	good performance	poor performance
calcium precursor	calcium nitrate, calcium gluconate	calcium carbonate/oxide
inert support	Y_2_O_3_, CeO_2_	Ca_12_Al_14_O_33_, CaZrO_3_
synthesis method	sol gel	surfactant template

optimal synthesis details	
CaO concentration (%)	70–90%
cycles	10–15
SMR reformer temperature	650 °C
SMR S/C ratio	6
carbonation time/temperature	15–20 min/650–700 °C
calcination time/temperature	5–10 min/900 °C
nickel wt %	2.5–10%
catalyst calcination temperature	600–700 °C
catalyst calcination time	10 h

## 4 Conclusions

In this work, a database for the prediction of Combined Sorbent Catalyst Material (CSCM) properties was developed through the application of data-mining, quantitative structure–property relationship analysis (QSPR), and multitask learning (MTL).

The development of the databases was discussed as well as the parameters chosen to produce accurate predictions for the properties of interest, last cycle capacity (g_sorbent_/g_CO_2__) and methane conversion (%). A thorough comparison of machine learning models, molecular descriptors, and their respective settings are also detailed. From the results, the application MTL was shown to improve the prediction capability of new molecules, compared to single-output models. Consequently, the MTL model was used to predict properties of unseen CSCM molecules somewhat effectively, 58% of the data resulting in an AARE of 50% or less.

Overall, this study has presented a methodology for the prediction of CSCM that has not been conducted before, with promising results. The study concludes that a combination of machine learning models and the application of molecular descriptors has the capability to predict the properties for a CSCM for the process of SE-SMR. The results from this aim to streamline and accelerate the experimental discovery of an optimal CSCM, by reducing the repetitive trial and error processes involved in material development. This study also possibly paves a route into expanding the QSPR approach for applications beyond drug discovery or biochemistry, which is the main use of molecular descriptors and QSPR.

Future directions for this study would be to use information from Table 5 to synthesize a new material using the model as is, then make changes to the models as appropriate, from the findings of this study, namely the reduction of experimental conditions. This would allow for, first, the synthesis of a material that would be assumed to possess the desired properties for a CSCM, but with the removal of the conditions, after this data are obtained, the prediction of how it would perform is assumed to be more accurate with fewer conditions specified. The material could then be synthesized and the performance compared against the models’ predictions, thus further validating this QSPR and MTL application for the process of SE-SMR. This could also pave a route for the application of multitask learning to be more utilized to predict the behavior of materials that rely on more than one type of property feature.

## Supporting Information Available

Databases and additional graphs (PDF)

## Notes

Data and Software Availability. Data regarding this work is available in the Supporting Information and is accessible from 10.17862/cranfield.rd.19241682. The prediction models are available in OCHEM platform (ochem.eu).

## Abbreviations

AD: Applicability Domain
ASNN: Associative Neural Network
CSCM: Combined Sorbent Catalyst Material
MAE: Mean Absolute Error
MTL: Multi-Task Learning
QSPR: Quantitative Structure–Property Relationship
RMSE: Root Mean Square Error
SE-SMR: Sorption-Enhanced Steam Methane Reforming
SMILES: Molecular-Input Line-Entry System
SMR: Steam Methane Reforming
STL: Single-Task Learning
