# Supplementary material for: Prediction of Combined Sorbent and Catalyst Materials for SE-SMR, Using QSPR and Multitask Learning
Source: Ind Eng Chem Res. 2022 Jun 23;61(26):9218–33. doi: 10.1021/acs.iecr.2c00971 (PMC9264356; doi:10.1021/acs.iecr.2c00971)
Supplement: Supplementary file 1 — ie2c00971_si_001.pdf [file ie2c00971_si_001.pdf]

Supporting Information

Prediction of combined sorbent and catalyst materials (CSCM) for SE-SMR, using QSPR and multi-task learning

*Paula Nkulikiyinka, Stuart Thomas Wagland, Vasilije Manovic and Peter T. Clough**

Energy and Power Theme, School of Water, Energy and Environment, Cranfield University, Cranfield, Bedfordshire, MK43 0AL, UK.

* Corresponding author: Peter Clough, p.t.clough@cranfield.ac.uk, +44 (0) 1234 754 873

Contents:

Table S1	Organization for Economic Co-operation and Development (OECD) guidelines for the development of a QSPR model
Table S2	Principal Component Analysis variance of the sorbent and catalyst data, indicating the relevance of the input conditions
Table S3	Feature importance calculated from PCA, showing the influential factor of the experimental conditions as inputs for the two databases
Table S4	Full sorbent database, with experimental conditions as inputs and last cycle capacity as the output property
Table S5	Full catalyst database, with experimental conditions as inputs and methane conversion as the output property
Table S6	Architecture of the ASNN model in OCHEM used to evaluate the STL vs MTL performance
Table S7	Architecture of the DNN model in OCHEM used to evaluate the STL vs MTL performance
Table S8	Architecture of the LSSVM model in OCHEM used to evaluate the STL vs MTL performance
Table S9	The combined sorbent and catalyst database gathered via datamining from literature. Data in black is input data added to the overall combined sorbent and catalyst database used to predict unseen molecules. Data in green is the unseen molecules
Table S10	The absolute relative error of the measured vs predicted methane conversion and last cycle capacity for unseen CSCM data using the final selected prediction model of ASNN and GSFrag descriptors
Figure S1	Actual vs Predicted scatter plot showing the effect of the cycle number on the Last cycle capacity (g/g)
Figure S2	Actual vs Predicted scatter plot showing the effect of the (a) carbonation time and (b) calcination time on the Last cycle capacity (g/g)
Figure S3	Actual vs Predicted scatter plot showing the effect of the (a) carbonation temperature and (b) calcination temperature on the Last cycle capacity (g/g)
Figure S4	Actual vs Predicted scatter plot showing the effect of the (a) SMR reformer temperature (ºC) and (b) BET Surface Area (m2/g) on the methane conversion (%)

Table S1. OECD guidelines for the development of a QSPR model

Guidelines as agreed by the Organization for Economic Co-operation and Development (OECD) member countries in November 2004, on the principles for validating QSAR/PR models. The agreed principles provide a basis for evaluating applicability of models and allow for more efficient assessment of chemical safety.

**Guideline**	**Examples/ Application**
**A defined endpoint**	An example of this is having consistency in the data used, for the calculation of the endpoint property a specific instance of this is if data is collected from multiple literature sources. As this was the case for this study, care was taken to ensure all data was inputted in heterogenous units, and with consideration regarding the type of experiment conducted.
**An unambiguous algorithm**	An indicator of a good QSPR model is the transferability of the model, i.e., the ability to replicate the descriptor values, the accuracy metrics and ability for a new user to predict new models using the developed model easily. In this case, it has been confirmed that all the models developed are readily reproducible with the given input data.
**A defined domain of applicability**	The applicability domain (AD) is defined as the physico-chemical, structural, or biological space, knowledge or information on which the training set of the model has been developed, and for which it is applicable to make predictions for new compounds' (1). Due to this its recommended that new data is predicted within the AD by interpolation as opposed to extrapolation. In this study the AD was well defined and further detail is given in Section 3.3.
**Appropriate measures of goodness**	Two examples of errors for this include use of excessive numbers of descriptors and failure to validate the model properly. Because of this, an evaluation of the number of descriptors was trialled.

**of fit, robustness and predictivity**	Furthermore, in accordance with the 'principle of parsimony', unnecessary additional descriptors were not utilised, as it often did not improve the fit. In terms of validation, its stated that use of external validation, i.e., using data not included in the training set is the only acceptable way to ensure good predictivity for a QSPR model.
**A mechanistic interpretation, if possible**	This can be validated by checking if the descriptors either have physiochemical interpretation that is consistent with a known mechanism or if data from literature can support the mechanistic basis of the model. In this case the latter was applied, in using experimental data from literature to validate the model against.

Table S2. PCA Variance

Principal Component Analysis variance of the sorbent and catalyst data, indicating the relevance of the input conditions

**Sorbent Variance**	
**PCA1**	21.5%
**PCA2**	15.7%
**PCA3**	14.3%
**PCA4**	12.2%
**PCA5**	8.5%
**PCA6**	7.5%
**PCA7**	6.2%
**PCA8**	4.7%
**PCA9**	4.0%
**PCA10**	3.2%
**PCA11**	2.3%
**Catalyst Variance**	
**PCA1**	32.0%
**PCA2**	20.3%
**PCA3**	15.7%
**PCA4**	13.3%
**PCA5**	11.6%
**PCA6**	7.1%

Table S3. Feature importance

Feature importance calculated from PCA, showing the influential factor of the experimental conditions as inputs for the two databases

	**Sorbent – Last cycle capacity (gCO_2/gSorbent)**
1	Precursor
2	CaO concentration (%)
3	Calcination temperature (°C)
4	Carbonation temperature (°C)
5	Synthesis Method
6	Calcination time(mins)
7	Initial cycle capacity (g/g)
8	Carbonation time (mins)
9	Cycle number
	**Catalyst – Methane conversion (%)**
1	Calcination temperature (°C)
2	SMR S/C ratio
3	BET surface area (m^2/g)
4	Nickel wt. %
5	SMR Reaction temperature (°C)
6	Time (h)

Table S4. Sorbent Database- Property- Last cycle CO2 capacity (g/g)

Full sorbent database, with experimental conditions as inputs and last cycle capacity as the output property

**Molecule**	CaO Concentration (%)	Calcium Precursor	Cycle Number	Method	Calcination Temperature (℃)	Calcination Time (mins)	Carbonation Temperature (℃)	Carbonation Time (mins)	Initial Cycle Capacity ($gCO_2/gSorbent$)	**Last cycle CO_2 capacity** ($gCO_2/gSorbent$)	**Ref**
$Ca_{12}Al_{14}O_{33}$	91	5	10	2	750	20	750	20	0.5562	0.5242	(2)
$Ca_{12}Al_{14}O_{33}$	82.16	4	30	2	750	20	750	20	0.5283	0.54	(3)
$Ca_{12}Al_{14}O_{33}$	80	8	7	1	800	10	650	30	0.523916	0.5	(4)
$Ca_{12}Al_{14}O_{33}$	90	4	30	2	750	20	750	20	0.5272	0.5556	(3)
$Ca_{12}Al_{14}O_{33}$	82	11	32	3	950	10	850	10	0.62	0.62	(5)
$Ca_{12}Al_{14}O_{33}$	82	11	100	3	700	10	700	10	0.39	0.39	(5)
$Ca_{12}Al_{14}O_{33}$	84	2	25	1	700	30	850	10	0.493344	0.42	(6)
$Ca_{12}Al_{14}O_{33}$	80	1	30	4	750	10	750	10	0.55704	0.374	(7)
$Ca_{12}Al_{14}O_{33}$	75	2	13	1	850	10	690	30	0.4	0.45	(8)
$Ca_{12}Al_{14}O_{33}$	55	11	100	3	950	10	850	10	0.4	0.4	(5)
$Ca_{12}Al_{14}O_{33}$	67	11	82	3	950	10	850	10	0.5	0.5	(5)
$Ca_{12}Al_{14}O_{33}$	67	5	10	2	750	20	750	20	0.503	0.4696	(2)
$Ca_{12}Al_{14}O_{33}$	34	1	50	1	800	5	650	40	0.258144	0.169598	(9)
$Ca_{12}Al_{14}O_{33}$	80	10	7	1	800	10	650	30	0.495815	0.44	(4)
$Ca_{12}Al_{14}O_{33}$	80	4	7	1	800	10	650	30	0.621946	0.6	(4)
$Ca_{12}Al_{14}O_{33}$	35	2	13	1	850	10	690	30	0.215659	0.21	(8)
$Ca_{12}Al_{14}O_{33}$	80	9	7	1	800	10	650	30	0.410043	0.36	[3]
$Ca_{12}Al_{14}O_{33}$	80	7	7	1	800	10	650	30	0.581804	0.57	[3]
$Ca_{12}Al_{14}O_{33}$	37	5	10	2	750	20	750	20	0.1467	0.1427	(2)
$Ca_{12}Al_{14}O_{33}$	58.35	11	100	2	950	10	850	10	0.36	0.25	[4]
$Ca_{12}Al_{14}O_{33}$	82	6	10	6	750	20	750	20	0.4421	0.3515	(10)
$Ca_{12}Al_{14}O_{33}$	75	2	56	1	980	5	650	30	0.52009	0.220173	[7]
$Ca_{12}Al_{14}O_{33}$	80	4	100	2	950	2.5	650	2.5	0.409	0.1282	(11)
$Ca_{12}Al_{14}O_{33}$	75	2	20	4	750	8.33333	750	8.33333	0.3009	0.2543	(12)
$Ca_{12}Al_{14}O_{33}$	52	5	10	2	750	20	750	20	0.213	0.1994	(2)

$Ca_{12}Al_{14}O_{33}$	75	2	200	1	1000	15	600	25	0.1518	0.0871	(13)
Al_2O_3	80	4	11	2	850	10	650	20	0.429264	0.43	(11)
$Ca_{12}Al_{14}O_{33}$	75	4	45	1	850	10	690	30	0.3256	0.2728	(14)
$Ca_{12}Al_{14}O_{33}$	82.16	3	30	2	900	10	650	30	0.3952	0.2562	(2)
$Ca_{12}Al_{14}O_{33}$	75	4	45	1	850	5	690	30	0.35	0.3	(15)
$Ca_{12}Al_{14}O_{33}$	75	2	13	1	950	10	690	30	0.42	0.33	[7]
Ca_2MnO_4	80	4	100	2	950	2.5	650	2.5	0.271	0.0845	(11)
TiO_2	71.85	1	40	4	750	10	600	10	0.18743	0.2363	(16)
$CaZrO_3$	70	3	30	4	950	0	650	15	0.3555	0.3075	(17)
$CaZrO_3$	23.83	11	1200	3	700	30	700	30	0.11	0.11	[4]
CeO_2	85	6	18	2	700	20	600	45	0.59	0.59	(18)
La_2O_3	80	4	100	2	950	2.5	650	2.5	0.3474	0.0614	(11)
MgO	58	4	50	1	758	30	758	30	0.45	0.43	(19)
MgO	75	1	51	1	850	6	650	10	0.3345	0.27	(20)
MgO	80	4	100	2	950	2.5	650	2.5	0.3923	0.1417	(11)
MgO	80	1	1250	8	750	30	750	20	0.36916	0.17	(21)
SiO_2	65.1	13	50	2	700	5	600	60	0.4861	0.3348	(22)
TiO_2	90	1	10	4	750	10	600	10	0.2	0.24	(16)
TiO_2	83.44	1	40	4	750	10	600	10	0.310737	0.187695	(16)
Y_2O_3	19.9	6	190	8	740	10	740	10	0.06	0.077	(23)
Y_2O_3	80	6	10	2	950	5	650	30	0.5426	0.4879	(24)
Yb_2O_3	90	4	50	4	900	5	650	25	0.4	0.22	(25)
Yb_2O_3	90	4	15	4	800	5	650	25	0.51	0.53	(25)
ZrO_2	90	3	10	2	900	10	650	20	0.57	0.36	[2]
ZrO_2	64	3	10	2	900	10	650	20	0.33	0.21	[2]
ZrO_2	80	3	10	2	900	10	650	20	0.49	0.31	[2]
ZrO_2	76.9	1	15	7	750	15	600	30	0.19	0.14	(26)
$CaZrO_3$	76.9	11	100	3	700	30	700	30	0.34	0.33	[4]
ZrO_2	60.3	11	50	8	750	10	750	10	0.31	0.15	(27)
$CaZrO_3$	66.7	11	100	3	950	10	850	10	0.21	0.21	[4]
ZrO_2	60.3	11	50	3	750	10	750	10	0.31	0.32	(27)

ZrO_2	60.3	11	23	3	950	0	850	10	0.25	0.23	(27)
$CaZrO_3$	66.7	11	100	3	700	30	700	30	0.23	0.23	[4]
ZrO_2	60.3	11	100	3	700	30	700	30	0.3	0.3	(27)
$Ca_{12}Al_{14}O_{33}$	85	4	45	1	850	5	690	30	0.45	0.36	(15)
Al_2O_3	91	6	30	2	750	20	750	20	0.52	0.55	[2]
CeO_2	91	6	18	2	700	20	600	45	0.55	0.535	(18)
CeO_2	80	6	18	2	700	20	600	45	0.57	0.49	(18)
CeO^2	95	6	18	2	700	20	600	45	0.44	0.45	(18)
Nd_2O_3	90	10	24	4	900	5	650	30	0.56	0.4	(25)
Nd_2O_3	60	10	24	4	900	5	650	30	0.46	0.31	(25)
Nd_2O_3	30	10	24	4	1000	5	650	30	0.23	0.22	(25)
$CaCO_3$	56	1	50	2	700	5	600	60	0.6683	0.277	(22)
SiO_2	50	13	50	2	700	5	600	60	0.2825	0.1835	(22)
SiO_2	75	13	50	2	700	5	600	60	0.5367	0.2985	(22)
CaO	100	1	50	2	700	5	600	60	0.5135	0.2675	(22)
$Ca_9Al_6O_{18}$	77.9	1	25	4	750	30	650	30	0.2626	0.3333	(26)
$Ca_9Al_6O_{18}$	59.2	1	25	4	750	30	650	30	0.1547	0.2033	(26)
$Ca_9Al_6O_{18}$	77.9	1	25	4	750	30	650	30	0.2371	0.2912	(26)
$Ca_9Al_6O_{18}$	59.2	1	25	4	750	30	650	30	0.2062	0.2599	(26)
$Ca_9Al_6O_{18}$	43.2	1	25	4	750	30	650	30	0.1661	0.2408	(26)
$Ca_9Al_6O_{18}$	29.3	1	25	4	750	30	650	30	0.1199	0.1867	(26)
Al_2O_3	91.7	1	25	4	750	30	650	30	0.2198	0.2624	(26)
MgO	93.3	1	25	4	750	30	650	30	0.4148	0.3054	(26)
MgO	87.4	1	25	4	750	30	650	30	0.405	0.3045	(26)
MgO	77.7	1	25	4	750	30	650	30	0.4015	0.2814	(26)
MgO	73.6	1	25	4	750	30	650	30	0.4235	0.275	(26)
MgO	63.5	1	25	4	750	30	650	30	0.41	0.2454	(26)
$CaCO_3$	56	1	25	4	750	30	650	30	0.265	0.2933	(26)
Y_2O_3	83.2	1	25	4	750	30	650	30	0.1701	0.2719	(26)
ZrO_2	76.9	1	25	4	750	30	650	30	0.2335	0.2168	(26)
$CaZrO_3$	73.8	1	25	4	750	30	650	30	0.3439	0.2893	(28)

$CaZrO_3$	55.6	1	25	4	750	30	650	30	0.2872	0.2595	(28)
$CaZrO_3$	42.2	1	25	4	750	30	650	30	0.2267	0.2033	(28)
$CaZrO_3$	31.9	1	25	4	750	30	650	30	0.1869	0.1823	(28)
MgO	77.7	1	25	4	930	10	650	30	0.4307	0.1907	(26)
$CaZrO_3$	73.8	1	25	4	930	10	650	30	0.3248	0.298	(28)
$Ca_9Al_6O_{18}$	77.9	1	25	4	930	10	650	30	0.3067	0.2273	(26)
$CaCO_3$	56	1	25	4	930	10	650	30	0.2683	0.1266	(26)
$Ca_9Al_6O_{18}$	77.9	1	25	4	930	10	650	30	0.2232	0.2107	(26)
Y_2O_3	80	6	10	2	850	5	650	30	0.5965	0.5399	(24)
Y_2O_3	80	6	10	2	950	5	650	30	0.5901	0.4974	(24)
Y_2O_3	80	6	10	2	850	5	650	30	0.5838	0.5748	(24)
Y_2O_3	80	6	10	2	1050	5	650	30	0.5843	0.3919	(24)
Y_2O_3	80	6	10	2	950	5	650	30	0.5721	0.5021	(24)
Y_2O_3	95	6	10	2	850	5	650	30	0.6058	0.5821	(24)
Y_2O_3	80	6	10	2	850	5	650	30	0.5847	0.5756	(24)
CaO	100	1	10	2	950	5	650	30	0.5292	0.4183	(24)
Y_2O_3	60	6	10	2	850	5	650	30	0.4365	0.4477	(24)
CaO	100	1	115	4	758	30	758	30	0.687016	0.178031	(29)
$MgAl_2O_4$	90	4	131	4	758	30	758	30	0.613301	0.45249	(29)
$MgAl_2O_4$	82	4	126	4	758	30	758	30	0.48762	0.437944	(29)
$MgAl_2O_4$	74	4	126	4	758	30	758	30	0.48762	0.442778	(29)
$MgAl_2O_4$	68	4	106	4	758	30	758	30	0.389737	0.405143	(29)
$MgAl_2O_4$	58	4	132	4	758	30	758	30	0.322062	0.34132	(29)
CaO	100	1	60	4	758	30	758	30	0.687016	0.209582	[28]
$MgAl_2O_4$	90	4	60	4	758	30	758	30	0.613301	0.540095	[28]
$MgAl_2O_4$	82	4	60	4	758	30	758	30	0.48762	0.478465	[28]
$MgAl_2O_4$	68	4	60	4	758	30	758	30	0.389737	0.410186	[28]
$MgAl_2O_4$	58	4	60	4	758	30	758	30	0.322062	0.344325	[28]
$Ca_{12}Al_{14}O_{33}$	87	2	50	1	850	5	700	30	0.448624	0.414285	[7]
$Ca_{12}Al_{14}O_{33}$	80	4	11	2	850	10	850	30	0.4696	0.4586	(11)
$Ca_{12}Al_{14}O_{33}$	66	1	50	1	800	5	650	40	0.25795	0.17097	(9)

$Ca_{12}Al_{14}O_{33}$	90	7	28	1	800	10	650	30	0.59	0.51	[3]
$Ca_3Al_2O_6$	91	1	50	5	850	10	650	30	0.6	0.41	(30)
$Ca_3Al_2O_6$	91	1	100	5	850	10	650	30	0.6	0.34	(30)
La_2O_3	80	4	11	2	850	10	850	30	0.4987	0.2197	(11)
La_2O_3	80	4	11	2	850	10	850	30	0.6009	0.5808	(11)
$Ca_{12}Al_{14}O_{33}$	80	4	11	2	850	10	850	30	0.4691	0.3188	(11)
$Ca_{12}Al_{14}O_{33}$	87	2	50	1	980	5	700	30	0.52	0.22	[7]
$Ca_3Al_2O_6$	74	1	20	3	850	10	700	30	0.4919	0.4248	[29]
$Ca_3Al_2O_6$	74	1	20	3	850	10	700	10	0.4739	0.3606	[29]
$Ca_3Al_2O_6$	74	1	20	3	850	10	700	5	0.4357	0.2805	[29]
$Ca_3Al_2O_6$	74	1	20	3	850	10	700	2	0.3258	0.1828	[29]
$Ca_3Al_2O_6$	70	1	50	3	850	10	700	30	0.4508	0.358	[29]
$Ca_3Al_2O_6$	74	1	50	3	850	10	700	30	0.4919	0.381	[29]
$Ca_3Al_2O_6$	78	1	50	3	850	10	700	30	0.5671	0.3186	[29]
$Ca_3Al_2O_6$	74	1	20	3	900	10	700	30	0.4858	0.3493	[29]
$Ca_3Al_2O_6$	74	1	20	3	950	10	700	30	0.4865	0.2969	[29]
$Ca_3Al_2O_6$	74	1	20	3	850	10	700	30	0.4927	0.4243	[29]
$Ca_3Al_2O_6$	74	1	20	3	950	10	700	30	0.4729	0.2799	[29]
$Ca_3Al_2O_6$	74	1	20	3	950	10	700	30	0.4593	0.2304	[29]
$Ca_3Al_2O_6$	74	1	20	3	850	10	680	30	0.5143	0.4343	[29]
$Ca_3Al_2O_6$	74	1	20	3	850	10	700	30	0.4929	0.4215	[29]
$Ca_3Al_2O_6$	74	1	20	3	850	10	725	30	0.4702	0.379	[29]
$Ca_3Al_2O_6$	74	1	20	3	850	10	650	30	0.4582	0.2916	[29]
$Ca_{12}Al_{14}O_{33}$	90	12	30	2	900	10	650	30	0.6827	0.624	[32]
$Ca_{12}Al_{14}O_{33}$	85	12	30	2	900	10	650	30	0.6417	0.6096	[32]
$Ca_{12}Al_{14}O_{33}$	75	12	30	2	900	10	650	30	0.5823	0.559	[32]
CaO	100	1	30	2	900	10	650	30	0.555	0.1277	[32]
$Ca_{12}Al_{14}O_{33}$	65	12	30	2	900	10	650	30	0.4891	0.4795	[32]
$Ca_{12}Al_{14}O_{33}$	90	12	30	2	950	10	650	30	0.6793	0.3638	[32]
$Ca_{12}Al_{14}O_{33}$	85	12	30	2	950	10	650	30	0.6407	0.4152	[32]
$Ca_{12}Al_{14}O_{33}$	75	12	30	2	950	10	650	30	0.5802	0.4401	[32]

CaO	100	1	30	2	950	10	650	30	0.552	0.089	[32]
$Ca_{12}Al_{14}O_{33}$	65	12	30	2	950	10	650	30	0.4898	0.4107	[32]
$Ca_9Al_6O_{18}$	80	10	35	2	900	10	650	30	0.5486	0.4665	[33]
$Ca_9Al_6O_{18}$	80	10	35	2	800	10	650	30	0.5482	0.5148	[33]
$Ca_9Al_6O_{18}$	80	10	35	2	1000	5	650	10	0.534	0.1933	[33]
$Ca_9Al_6O_{18}$	80	10	35	2	800	10	650	30	0.5272	0.5244	[33]
$Ca_9Al_6O_{18}$	80	10	35	2	900	10	650	10	0.5185	0.326	[33]
MgO	75	8	24	4	900	10	650	10	0.5758	0.5373	[34]
MgO	67	8	24	4	900	10	650	10	0.499	0.4806	[34]
CaO	100	1	24	4	900	10	650	10	0.4863	0.1891	[34]
MgO	52	8	24	4	900	10	650	10	0.391	0.3725	[34]
$Ca_9Al_6O_{18}$	92.5	4	31	2	800	10	700	30	0.587734	0.565443	[35]
$Ca_9Al_6O_{18}$	92.5	4	31	2	800	10	650	30	0.553211	0.56465	[35]
$Ca_9Al_6O_{18}$	92.5	4	31	2	800	10	600	30	0.460751	0.431713	[35]
$Ca_9Al_6O_{18}$	92.5	4	31	2	800	10	650	30	0.55382	0.564516	[35]
$Ca_9Al_6O_{18}$	92.5	4	31	2	850	10	650	30	0.552235	0.524109	[35]
$Ca_9Al_6O_{18}$	92.5	4	31	2	900	10	650	30	0.58472	0.511036	[35]
$Ca_9Al_6O_{18}$	80	4	31	2	930	5	650	30	0.519672	0.334915	[35]
$CaZrO_3$	90	3	30	4	800	0	650	15	0.3101	0.3645	[17]
$CaZrO_3$	82	3	30	4	800	0	650	15	0.2503	0.3113	[17]
$CaZrO_3$	70	3	30	4	800	0	650	15	0.1743	0.2664	[17]
$CaZrO_3$	90	3	30	4	950	0	650	15	0.4622	0.2992	[17]
$CaZrO_3$	82	3	30	4	950	0	650	15	0.4344	0.3087	[17]
TiO_2	90	1	10	4	750	10	600	10	0.206206	0.23313	[16]
TiO_2	85	1	10	4	750	10	600	10	0.183203	0.159056	[16]
TiO_2	92	1	10	4	750	10	600	10	0.172168	0.163632	[16]
TiO_2	80	1	10	4	750	10	600	10	0.168648	0.159408	[16]
TiO_2	95	1	10	4	750	10	600	10	0.113714	0.120908	[16]
$Ca_{12}Al_{14}O_{33}$	82.16	3	30	2	900	10	650	30	0.4918	0.3088	[2]
MgO	85	8	24	4	900	10	650	10	0.6282	0.5693	[34]
MgO	95	8	24	4	900	10	650	10	0.6577	0.5589	[34]

$Ca_{12}Al_{14}O_{33}$	85	2	21	4	750	8.33333	750	8.33333	0.0812	0.2105	[12]
MgO	25	2	21	4	750	8.33333	750	8.33333	0.0665	0.0636	[12]
$Ca_{12}Al_{14}O_{33}$	75	2	20	4	750	8.33333	750	8.33333	0.043	0.0636	[12]
$Ca_{12}Al_{14}O_{33}$	75	2	20	4	750	8.33333	750	8.33333	0.043	0.079	[12]
MgO	50	2	21	4	750	8.33333	750	8.33333	0.0323	0.0276	[12]
$Ca_{12}Al_{14}O_{33}$	50	2	21	4	750	8.33333	750	8.33333	0.0125	0.0338	[12]
MgO	63	2	21	4	750	8.33333	750	8.33333	0.0724	0.0816	[12]
$Ca_{12}Al_{14}O_{33}$	80	3	30	6	800	5	650	10	0.499695	0.298191	[36]
$Ca_{12}Al_{14}O_{33}$	80	3	30	6	900	5	650	10	0.472178	0.254909	[36]
$Ca_{12}Al_{14}O_{33}$	80	3	30	6	900	5	650	10	0.428466	0.189125	[36]
$Ca_{12}Al_{14}O_{33}$	80	3	30	6	900	5	650	10	0.410982	0.213346	[36]
$Ca_{12}Al_{14}O_{33}$	95	3	30	6	900	5	650	10	0.549851	0.164672	[36]
$Ca_{12}Al_{14}O_{33}$	90	3	30	6	900	5	650	10	0.53663	0.205625	[36]
$Ca_{12}Al_{14}O_{33}$	85	3	30	6	900	5	650	10	0.498256	0.22465	[36]
$Ca_{12}Al_{14}O_{33}$	80	3	30	6	900	5	650	10	0.428765	0.189985	[36]
$Ca_{12}Al_{14}O_{33}$	75	3	30	6	900	5	650	10	0.397003	0.164672	[36]
$Ca_{12}Al_{14}O_{33}$	85	3	15	6	900	5	650	10	0.412839	0.243346	[36]
$Ca_{12}Al_{14}O_{33}$	75	3	15	6	900	5	650	10	0.36416	0.20554	[36]
$Ca_{12}Al_{14}O_{33}$	75	3	18	4	900	10	650	30	0.5764	0.483	[37]
$Ca_{12}Al_{14}O_{33}$	75	3	18	4	900	10	650	30	0.4963	0.3239	[37]
$Ca_{12}Al_{14}O_{33}$	75	3	18	4	900	10	650	30	0.4295	0.2579	[37]
$Ca_{12}Al_{14}O_{33}$	75	1	18	4	900	10	650	30	0.4188	0.1885	[37]
$Ca_{12}Al_{14}O_{33}$	75	10	18	4	900	10	650	30	0.4772	0.3966	[37]
$Ca_{12}Al_{14}O_{33}$	75	4	18	4	900	10	650	30	0.4705	0.3611	[37]
$Ca_{12}Al_{14}O_{33}$	75	3	18	4	900	10	650	30	0.4682	0.2841	[37]
$Ca_{12}Al_{14}O_{33}$	75	8	18	4	900	10	650	30	0.4283	0.3881	[37]
$Ca_{12}Al_{14}O_{33}$	75	9	18	4	900	10	650	30	0.4184	0.2785	[37]
$Ca_{12}Al_{14}O_{33}$	75	4	18	4	900	10	650	30	0.4028	0.1771	[37]
$Ca_{12}Al_{14}O_{33}$	75	4	18	4	900	10	650	30	0.3736	0.1543	[37]
$Ca_{12}Al_{14}O_{33}$	75	3	62	4	900	10	650	30	0.5764	0.3406	[37]
$Ca_{12}Al_{14}O_{33}$	75	10	68	4	900	10	650	30	0.4779	0.3632	[37]

$Ca_{12}Al_{14}O_{33}$	75	8	66	4	900	10	650	30	0.4298	0.3099	[37]
$Ca_{12}Al_{14}O_{33}$	50	3	50	4	900	10	650	30	0.3315	0.2735	[37]
$Ca_{12}Al_{14}O_{33}$	50	10	70	4	900	10	650	30	0.2719	0.2486	[37]
$Ca_{12}Al_{14}O_{33}$	50	8	66	4	900	10	650	30	0.2068	0.2048	[37]
Al_2O_3	90	4	11	2	850	10	650	20	0.461639	0.366498	[11]
Al_2O_3	60	4	11	2	850	10	650	20	0.384814	0.462	[11]
$Ca_{12}Al_{14}O_{33}$	91	4	10	2	750	20	750	20	0.6158	0.4625	[2]
$Ca_{12}Al_{14}O_{33}$	91	4	10	2	750	20	750	20	0.5516	0.5215	[2]
$Ca_{12}Al_{14}O_{33}$	91	1	10	2	750	20	750	20	0.0423	0.0897	[2]
$Ca_{12}Al_{14}O_{33}$	91	4	30	2	750	20	750	20	0.6188	0.3099	[2]
$Ca_{12}Al_{14}O_{33}$	91	5	30	2	750	20	750	20	0.5537	0.5067	[2]
$Ca_{12}Al_{14}O_{33}$	82	4	10	6	750	20	750	20	0.5385	0.2976	[10]
$Ca_{12}Al_{14}O_{33}$	82	4	10	6	750	20	750	20	0.4198	0.2897	[10]
$Ca_{12}Al_{14}O_{33}$	82	1	10	6	750	20	750	20	0.5377	0.2983	[10]
$Ca_{12}Al_{14}O_{33}$	82	6	10	6	750	20	750	20	0.5215	0.2131	[10]
$Ca_{12}Al_{14}O_{33}$	82	4	10	6	750	20	750	20	0.4853	0.3461	[10]
$Ca_3Al_2O_6$	75	2	30	1	850	5	700	30	0.4479	0.4346	[7]
$Ca_3Al_2O_6$	75	2	30	1	850	5	650	30	0.3617	0.46	[7]
$Ca_3Al_2O_6$	75	2	15	1	850	5	650	30	0.1746	0.3091	[7]
$Ca_3Al_2O_6$	75	2	15	1	850	5	650	30	0.0482	0.1867	[7]
$Ca_3Al_2O_6$	75	2	15	1	850	5	650	30	0.0341	0.1391	[7]
$CaZrO_3$	62.7	1	15	7	750	30	600	30	0.193194	0.12817	[28]
$CaZrO_3$	42	1	15	7	750	30	600	30	0.190553	0.134856	[28]
$CaZrO_3$	78.2	1	15	7	750	30	600	30	0.166702	0.130234	[28]
$CaZrO_3$	13	1	15	7	750	30	600	30	0.136413	0.095735	[28]
$CaZrO_3$	42	1	15	7	750	30	700	30	0.245857	0.247321	[28]
$CaZrO_3$	42	1	15	7	750	30	650	30	0.215933	0.200011	[28]
$CaZrO_3$	42	1	15	7	750	30	600	30	0.190568	0.134032	[28]

Table S5. Catalyst Database- Property- Methane Conversion (%)

Full catalyst database, with experimental conditions as inputs and methane conversion as the output property

Molecule	Nickel wt. %	Calcination temperature catalyst	Reaction temperature (ºC)	S/C ratio	Time (h)	BET Surface Area (m^2/g)	Methane Conversion (%)	Ref
$SiO_2Al_2O_3$	4.34	900	650	1	50	105.8	0.075663	38
$SiO_2Al_2O_3$	4.34	900	700	1	50	105.8	0.049667	38
$SiO_2Al_2O_3$	4.34	900	750	1	50	105.8	0.985249	38
$SiO_2Al_2O_3$	4.34	900	800	1	50	105.8	0.991883	38
$SiO_2Al_2O_3$	4.34	900	850	1	50	105.8	1	38
$SiO_2Al_2O_3$	4.34	900	900	1	50	105.8	0.996566	38
$SiO_2Al_2O_3$	6.2	900	650	1	50	73.4	0.00011	38
$SiO_2Al_2O_3$	6.2	900	700	1	50	73.4	0.001592	38
$SiO_2Al_2O_3$	6.2	900	750	1	50	73.4	0.743141	38
$SiO_2Al_2O_3$	6.2	900	800	1	50	73.4	0.859668	38
$SiO_2Al_2O_3$	6.2	900	850	1	50	73.4	0.905794	38
$SiO_2Al_2O_3$	6.2	900	900	1	50	73.4	0.97596	38
$MgAl_2O_4$	15.3	350	600	5	9	70.2	0.419067375	39
$MgAl_2O_4$	15.3	500	600	5	9	64.8	0.44841325	39
$MgAl_2O_4$	15.3	650	600	5	9	50.2	0.4800525	39
$MgAl_2O_4$	15.3	850	600	5	9	43.2	0.485568	39
$MgAl_2O_4$	15.3	1000	600	5	9	42.3	0.486923375	39
$MgAl_2O_4$	15.3	350	600	5	9	70.2	0.32975025	39
$MgAl_2O_4$	15.3	500	600	5	9	64.8	0.3691085	39
$MgAl_2O_4$	15.3	650	600	5	9	50.2	0.410592625	39
$MgAl_2O_4$	15.3	850	600	5	9	43.2	0.418064	39
$MgAl_2O_4$	15.3	1000	600	5	9	42.3	0.399417	39
$MgAl_2O_4$	15.3	350	600	5	9	70.2	0.296565125	39
$MgAl_2O_4$	15.3	500	600	5	9	64.8	0.3596845	39
$MgAl_2O_4$	15.3	650	600	5	9	50.2	0.342117125	39
$MgAl_2O_4$	15.3	850	600	5	9	43.2	0.379053875	39
$MgAl_2O_4$	15.3	1000	600	5	9	42.3	0.348423375	39
Al_2O_4	34	1000	700	2.4	12	37	0.78	40

$NiAl_2O_5$	45	1000	700	2.4	12	25	0.82	[40]
Al_2O_3	10	700	600	3	30	93	0.440502	[41]
Al_2O_3	10	700	650	3	30	93	0.638497	[41]
Al_2O_3	10	700	700	3	30	93	0.792961	[41]
Al_2O_3	10	700	750	3	30	93	0.846399	[41]
Al_2O_3	10	700	800	3	30	93	0.871973	[41]
$Y_2Zr_2O_7$	9.7	800	550	2	0.5	1.5	0.211503	[42]
$Y_2Zr_2O_7$	9.7	800	600	2	0.5	1.5	0.340291	[42]
$Y_2Zr_2O_7$	9.7	800	650	2	0.5	1.5	0.462472	[42]
$Y_2Zr_2O_7$	9.7	800	700	2	0.5	1.5	0.597867	[42]
$Y_2Zr_2O_7$	9.7	800	750	2	0.5	1.5	0.698571	[42]
$Y_2Zr_2O_7$	9.7	800	800	2	0.5	1.5	0.749708	[42]
$Y_2Zr_2O_7$	9.8	800	550	2	0.5	20.5	0.323846	[42]
$Y_2Zr_2O_7$	9.8	800	600	2	0.5	20.5	0.437764	[42]
$Y_2Zr_2O_7$	9.8	800	650	2	0.5	20.5	0.614463	[42]
$Y_2Zr_2O_7$	9.8	800	700	2	0.5	20.5	0.845681	[42]
$Y_2Zr_2O_7$	9.8	800	750	2	0.5	20.5	0.926558	[42]
$Y_2Zr_2O_7$	9.8	800	800	2	0.5	20.5	0.990913	[42]
$Y_2Zr_2O_7$	9.8	800	550	2	0.5	62.6	0.261065	[42]
$Y_2Zr_2O_7$	9.8	800	650	2	0.5	62.6	0.592986	[42]
$Y_2Zr_2O_7$	9.8	800	700	2	0.5	62.6	0.77464	[42]
$Y_2Zr_2O_7$	9.8	800	750	2	0.5	62.6	0.901776	[42]
$Y_2Zr_2O_7$	9.8	800	800	2	0.5	62.6	0.949612	[42]
$Y_2Zr_2O_7$	9.8	800	800	2	100	20.5	0.990792667	[42]
$Y_2Zr_2O_7$	9.8	800	800	2	100	62.6	0.89036	[42]
$Y_2Zr_2O_7$	9.7	800	800	2	100	1.5	0.661074	[42]
$Y_2Zr_2O_7$	9.8	800	800	2	200	20.5	0.63712775	[42]
$Y_2Zr_2O_7$	9.8	800	800	2	90	62.6	0.545453667	[42]
$Y_2Zr_2O_7$	9.7	800	800	2	90	1.5	0.294691333	[42]
$Al_2O_3.C_4H_8O_2$	7.2	700	550	2.2	16	257	0.611779333	[43]
$Al_2O_3.C_4H_8O_2$	7.6	700	550	2.2	16	276	0.625771333	[43]
$Al_2O_3.C_4H_8O_2$	13	700	550	2.2	16	265	0.662265	[43]
$Al_2O_3.C_4H_8O_2$	11.9	700	550	2.2	16	262	0.639041667	[43]

$Al_2O_3.C_4H_8O_2$	8.8	700	550	2.2	16	274	0.634857667	[43]
$Y_2Ti_2O_7$	7	800	600	2	0.5	21	0.509268	[44]
$Y_2Ti_2O_7$	7	800	650	2	0.5	21	0.683814	[44]
$Y_2Ti_2O_7$	7	800	700	2	0.5	21	0.826027	[44]
$Y_2Ti_2O_7$	7	800	750	2	0.5	21	0.914368	[44]
$Y_2Ti_2O_7$	7	800	800	2	0.5	21	0.96499	[44]
$Y_2Sn_2O_7$	6.9	800	600	2	0.5	38	0.01718	[44]
$Y_2Sn_2O_7$	6.9	800	650	2	0.5	38	0.039073	[44]
$Y_2Sn_2O_7$	6.9	800	750	2	0.5	38	0.127747	[44]
$Y_2Sn_2O_7$	6.9	800	800	2	0.5	38	0.165798	[44]
$Y_2Zr_2O_7$	7	800	600	2	0.5	2	0.34404	[44]
$Y_2Zr_2O_7$	7	800	650	2	0.5	2	0.500623	[44]
$Y_2Zr_2O_7$	7	800	700	2	0.5	2	0.660801	[44]
$Y_2Zr_2O_7$	7	800	750	2	0.5	2	0.786855	[44]
$Y_2Zr_2O_7$	7	800	800	2	0.5	2	0.896742	[44]
$Y_2Ce_2O_7$	7.1	800	600	2	0.5	4	0.387142	[44]
$Y_2Ce_2O_7$	7.1	800	700	2	0.5	4	0.673375	[44]
$Y_2Ce_2O_7$	7.1	800	750	2	0.5	4	0.797629	[44]
$Y_2Ce_2O_7$	7.1	800	800	2	0.5	4	0.8734	[44]
$Y_2Ti_2O_7$	6.9	800	750	2	50	21	0.814813	[44]
$Y_2Ce_2O_7$	7.1	800	750	2	50	4	0.604116	[44]
$Y_2Zr_2O_7$	7	800	750	2	50	2	0.401646	[44]
$Y_2Sn_2O_7$	6.9	800	750	2	50	38	0.07572	[44]
$La_2Ti_2O_7$	7.1	800	600	2	0.5	19.2	0.412934	[45]
$Pr_2Ti_2O_7$	6.9	800	600	2	0.5	18.5	0.371955	[45]
$Sm_2Ti_2O_7$	7.1	800	600	2	0.5	16.9	0.445095	[45]
$Y_2Ti_2O_7$	7	800	600	2	0.5	15.8	0.450934	[45]
$La_2Ti_2O_7$	7.1	800	650	2	0.5	19.2	0.559158	[45]
$Pr_2Ti_2O_7$	6.9	800	650	2	0.5	18.5	0.544514	[45]
$Y_2Ti_2O_7$	7	800	650	2	0.5	15.8	0.623494	[45]
$La_2Ti_2O_7$	7.1	800	700	2	0.5	19.2	0.695141	[45]
$Pr_2Ti_2O_7$	6.9	800	700	2	0.5	18.5	0.680497	[45]

$Sm_2Ti_2O_7$	7.1	800	700	2	0.5	16.9	0.756562	[45]
$Y_2Ti_2O_7$	7	800	700	2	0.5	15.8	0.822388	[45]
$La_2Ti_2O_7$	7.1	800	750	2	0.5	19.2	0.804787	[45]
$Sm_2Ti_2O_7$	7.1	800	750	2	0.5	16.9	0.876452	[45]
$Y_2Ti_2O_7$	7	800	750	2	0.5	15.8	0.910089	[45]
$La_2Ti_2O_7$	7.1	800	800	2	0.5	19.2	0.886637	[45]
$Pr_2Ti_2O_7$	6.9	800	800	2	0.5	18.5	0.911496	[45]
$Sm_2Ti_2O_7$	7.1	800	800	2	0.5	16.9	0.934893	[45]
$Y_2Ti_2O_7$	7	800	800	2	0.5	15.8	0.964139	[45]
$Pr_2Ti_2O_7$	6.9	800	800	2	50	18.5	0.805779	[45]
$La_2Ti_2O_7$	7.1	800	800	2	50	19.2	0.749774	[45]
$Sm_2Ti_2O_7$	7.1	800	800	2	50	16.9	0.644985	[45]
$Y_2Ti_2O_7$	7	800	800	2	50	15.8	0.616078	[45]
Al_2O_3	10	550	600	1.2	1	101	0.502171	[46]
Al_2O_3	10	550	650	1.2	1	101	0.833715	[46]
Al_2O_3	10	550	700	1.2	1	101	0.934697	[46]
Al_2O_3	10	550	650	1.2	1	205	0.881292	[46]
Al_2O_3	10	550	700	1.2	1	205	0.952997	[46]
Al_2O_3	10	550	600	1.2	20	101	0.532417	[46]
Al_2O_3	10	550	600	1.2	5	101	0.484269	[46]
Al_2O_3	10	550	650	1.2	20	101	0.855784	[46]
Al_2O_3	10	550	650	1.2	5	101	0.842772	[46]
Al_2O_3	10	550	700	1.2	20	101	0.912283	[46]
Al_2O_3	10	550	700	1.2	5	101	0.954138	[46]
Al_2O_3	10	550	600	1.2	5	205	0.632041	[46]
Al_2O_3	10	550	600	1.2	20	205	0.636707	[46]
Al_2O_3	10	550	650	1.2	5	205	0.860525	[46]
Al_2O_3	10	550	700	1.2	5	205	0.990004	[46]
Al_2O_3	10	550	700	1.2	20	205	0.98705	[46]
Al_2O_3	10	550	600	3.5	5	101	0.849106	[46]
Al_2O_3	10	550	600	3.5	20	101	0.860623	[46]
Al_2O_3	10	550	650	3.5	5	101	0.9405	[46]
Al_2O_3	10	550	650	3.5	20	101	0.980956	[46]

Al_2O_3	10	550	700	3.5	20	101	1	[46]
Al_2O_3	10	550	600	6.1	5	101	0.966395	[46]
Al_2O_3	10	550	600	6.1	20	101	0.980963	[46]
Al_2O_3	10	550	650	6.1	5	101	0.996862	[46]
Al_2O_3	10	550	650	6.1	20	101	1	[46]
Al_2O_3	10	550	700	6.1	5	101	0.996862	[46]
Al_2O_3	10	550	700	6.1	20	101	1	[46]
$CaZrO_3.Al_2O_3$	10	750	700	1	1	7.4117	0.300809	[47]
$CaZrO_3.Al_2O_3$	10	750	700	1	10	7.4117	0.302993	[47]
$CaZrO_3.Al_2O_3$	10	750	700	1	1	8.8919	0.637843	[47]
$CaZrO_3.Al_2O_3$	10	750	700	1	10	8.8919	0.630293	[47]
Al_2O_3	10	750	700	1	1	4.067	0.685295	[47]
Al_2O_3	10	750	700	1	10	4.067	0.641244	[47]
$CaZrO_3.Al_2O_3$	10	750	700	1	1	10.7274	0.684079	[47]
$CaZrO_3.Al_2O_3$	10	750	700	1	10	10.7274	0.669228	[47]
Al_2O_3	10	750	700	0.33	1	4.067	0.154028	[47]
Al_2O_3	10	750	700	0.33	10	4.067	0.126861	[47]
$CaZrO_3.Al_2O_3$	10	750	700	0.33	1	10.7274	0.183494	[47]
$CaZrO_3.Al_2O_3$	10	750	700	0.33	10	10.7274	0.188356	[47]
$CaZrO_3.Al_2O_3$	10	750	700	3	1	10.7274	0.661359	[47]
$CaZrO_3.Al_2O_3$	10	750	700	3	10	10.7274	0.667502	[47]
Al_2O_3	10	750	700	3	1	4.067	0.813815	[47]
$CaZrO_3.Al_2O_3$	10	750	700	0.33	9	8.8919	0.207896	[47]
$CaZrO_3.Al_2O_3$	10	750	700	0.33	97	8.8919	0.216223	[47]
Al_2O_3	10	750	700	0.33	8	4.067	0.141751	[47]
Al_2O_3	10	750	700	0.33	96	4.067	0.024964	[47]
$MgAl_2O_4$	17.9	900	750	1.24	1	64	0.027388	[48]
$MgAl_2O_4$	17.9	900	800	1.24	1	64	0.274466	[48]
$MgAl_2O_4$	17.9	900	850	1.24	1	64	0.388216	[48]
$MgAl_2O_4$	12.3	900	750	1.24	1	31	0.094834	[48]
$MgAl_2O_4$	12.3	900	800	1.24	1	31	0.337217	[48]
$MgAl_2O_4$	12.3	900	850	1.24	1	31	0.599987	[48]
$MgAl_2O_4$	13.4	900	750	1.24	1	71	0.279938	[48]

$MgAl_2O_4$	13.4	900	800	1.24	1	71	0.437609	[48]
$MgAl_2O_4$	13.4	900	850	1.24	1	71	0.749001	[48]
CeO_2-ZrO_2	10	500	500	1.94	5	63.6	0.099	[49]
CeO_2-ZrO_2	10	500	600	1.94	5	63.6	0.292	[49]
CeO_2-ZrO_2	10	500	700	1.94	5	63.6	0.458	[49]
CeO_2-ZrO_2	10	500	800	1.94	5	63.6	0.666	[49]
Al_2O_3	10	500	700	1.94	5	123	0.338	[49]
Al_2O_3	10	500	800	1.94	5	123	0.711	[49]
$LaAlO_3$	10	500	800	2	0.5	15.9	0.918695	[50]
$LaAlO_3$	10	500	800	2	1	15.9	0.914169	[50]
$LaAlO_3$	10	500	800	2	5	15.9	0.918412	[50]
$SrTiO_3$	10	500	800	2	0.5	13.4	0.878246	[50]
$SrTiO_3$	10	500	800	2	5	13.4	0.88545	[50]
$LaFeO_3$	10	500	800	2	1	15.9	0.370349	[50]
$LaFeO_3$	10	500	800	2	5	15.9	0.371596	[50]
$BaTiO_3$	10	500	800	2	0.5	15	0.06027	[50]
$BaTiO_3$	10	500	800	2	1	15	0.081212	[50]
$BaTiO_3$	10	500	800	2	5	15	0.016542	[50]
$LaAlO_3$	10	500	800	1	17	15.9	0.833139	[50]
$LaAlO_3$	10	500	800	1	23	15.9	0.803568	[50]
$RuAl_2O_3$	20	900	650	1.2	0.5	1.05	0.253151	[51]
Al_2O_3	20	900	650	1.2	0.5	0.32	0.224613	[51]
$RuAl_2O_3$	20	900	650	1.2	9.5	1.05	0.280361	[51]
Al_2O_3	20	900	650	1.2	9.5	0.32	0.20901	[51]

Filters of descriptors

Additional filter settings in the OCHEM platform

- Eliminate descriptors with less than 2 unique values
- Delete descriptors that have absolute values larger than 999999
- Delete descriptors that have variance smaller than 0.01
- Group descriptors, that have pair-wise correlations Pearson's correlation coefficient R larger than 0.95

Table S6. Architecture of the ASNN model in OCHEM

Setting	*Value*
Training method	SuperSAB
Number of neurons in hidden layer	3
Learning iterations	1000
Ensemble	64
Additional Parameters	PARTITION=3, SELECTION=2

Table S7. Architecture of the DNN model in OCHEM

Setting	*Value*
Epochs	1024
Batch size	10000
Training/internal validation set ratio	0.8
Model	Dense7 (seven layers) Net
Optimisation method	Adam
Activation function	relu

Table S8. Architecture of the LSSVM model in OCHEM

Setting	*Value*
Kernel	RBF Kernel
Internal CV (cross validation) folds	5

Table S9. CSCM database

Experimental conditions as inputs and last cycle capacity and methane conversion as outputs, as gathered from literature for CSCM. Data in black, is input data added to the overall combined sorbent (239) and catalyst (183) database used to predict unseen molecules (24) (446 datapoints overall for MTL). Data in green is the unseen molecules (17).

`	Molecule	Nickel wt. %	Calcination Temperature Catalyst (ºC)	Reaction Temperature (ºC)	S/C Ratio	Time (h)	BET Surface Area (m^2/g)	Methane Conversion (%)	CaO Concentration (%)	Cycle Number	Calcium Precursor	Method	Calcination Temperature (ºC)	Calcination Time (mins)	Carbonation Temperature (ºC)	Carbonation Time (mins)	Initial Cycle Capacity ($gCO_2/gSorbent$)	Last Cycle CO_2 Capacity ($gCO_2/gSorbent$)	Reference
1	$Ca_5Al_6O_{14}$	15	850	650	2	2		0.95	61.7	50	10	2	800	10	650	30	0.484	0.449	[52]
2	$CaZrO_3$	15	850	650	3	1	12.8	0.83	60.3	50	6	2	800	10	650	20	0.399	0.355	[53]
3	$Ca_{12}Al_{14}O_{33}$	10	900	600	3	1	7.1	0.93	30	200	2	4	900	30	600	30	0.206	0.192	[54]
4	$Ca_{12}Al_{14}O_{33}$	5.2	800	650	4	0.67	10.5	0.97	18	200	3	4	925	15	650	20	0.116	0.028	[55]
5	$Ca_5Al_6O_{14}$	15	850	650	3	0.83	17.5	0.94	18	40	10	2	800	45	650	30	0.130	0.115	[56]
6	$Ca_{12}Al_{14}O_{33}$	3	900	650	3	3	12.7	0.10	54	50	4	4	900	60	650	60	0.195	0.063	[57]
7	$Ca_{12}Al_{14}O_{33}$	3	900	650	3	3	9.2	0.12	30	50	4	4	900	60	650	60	0.136	0.127	[57]
8	$Ca_{12}Al_{14}O_{33}$	3.6	750	650	3	50	11.5	0.99	75	30	6	3	800	10	650	30	0.450	0.338	[58]
9	$Ca_{12}Al_{14}O_{33}$	3.6	750	650	3	50	12.6	0.80	75	30	1	4	800	10	650	30	0.401	0.292	[58]
10	$Ca_{12}Al_{14}O_{33}$	13.2	900	650	3	0.5	12.44	0.96	10	200	2	8	850	10	650	10	0.104	0.099	[59]
11	$Ca_{12}Al_{14}O_{33}$	20	900	650	3.4	1	9.5	0.36	70	44	4	1	850	5	690	30	0.303	0.264	[60]
12	$Ca_{12}Al_{14}O_{33}$	16	900	650	3.4	1	8.8	0.81	70	44	4	1	850	5	690	30	0.292	0.307	[60]
13	$Ca_{12}Al_{14}O_{33}$	11	900	650	3.4	1	10	0.59	70	45	4	1	850	5	690	30	0.317	0.271	[60]
14	$Ca_{12}Al_{14}O_{33}$	8	900	650	3.4	1	11	0.22	70	44	4	1	850	5	690	30	0.378	0.281	[60]
15	$CaZrO_3$	3.4	900	600	3	3	17.7	0.70	95.4	10	2	4	850	30	600	30	0.308	0.287	[61]
16	CeO_2	2.5	900	600	3	3	8.2	0.61	96.3	10	2	4	850	30	600	30	0.226	0.236	[61]
17	La_2O_3	2.8	900	600	3	3	13.1	0.46	92.6	10	2	4	850	30	600	30	0.295	0.247	[61]
18	Al_2O_3	3	900	650	3	1	10.1	0.96	29	50		4	800	30	650	30	0.099	0.087	[62]

19	Al_2O_3	3	900	650	3	1	9.2	0.96	29	50		4	800	30	650	30	0.135	0.123	62
20	Al_2O_3	3	900	650	3	1	14.9	0.96	52	50		4	800	30	650	30	0.257	0.097	62
21	Al_2O_3	3	900	650	3	1	12.7	0.96	52	50		4	800	30	650	30	0.194	0.061	62
22	Al_2O_3	15	900	650	4	2	9.9		56	10	10	2	800	10	650	30	0.218	0.233	63
23	Al_2O_3	15	900	650	4	2	11.5		68	10	10	2	800	10	650	30	0.396	0.397	63
24	Al_2O_3	15	900	650	4	2	12.1		72.3	10	10	2	800	10	650	30	0.457	0.456	63
25	Al_2O_3	15	900	650	4	2	12.3		75	10	10	2	800	10	650	30	0.492	0.487	63
26	Al_2O_3	15	900	650	4	2	10.9		77.2	10	10	2	800	10	650	30	0.510	0.512	63
27	Al_2O_3	15	900	650	4	2	9.6		78.5	10	10	2	800	10	650	30	0.525	0.526	63
28	Al_2O_3	15	900	650	2	2	12.1		72.3	50	10	2	800	10	650	30	0.395	0.376	63
29	$CaZrO_3$	18	800	650	4	2	15.9	0.97	34.3	10	4	4	800	20	650	30			64
30	$CaZrO_3$	20.5	800	650	4	2	9.6	0.99	33.2	10	4	4	800	20	650	30			64
31	$CaZrO_3$	13	800	650	4	2	8.3	0.89	36.4	2	4	4	800	20	650	30			64
32	$Ca_9Al_6O_{18}$	25	900	650	4	2	10	0.99	58.4	10	1	1	800	20	650	30			65
33	$Ca_9Al_6O_{18}$	18	900	650	4	2	12.9	0.96	63.9	10	1	1	800	20	650	30			65
34	$La_2O_3Al_2O_3$		550	600	4	5	29.18	0.78		30	1	8	725	10	600	10	0.136	0.053	66
35	$La_2O_3Al_2O_3$		550	600	4	5	33.37			30	1	8	725	10	600	10	0.091	0.037	66
36	$MgAl_2O_4Ca_{12}Al_{14}O_{33}$	5.3	800	650	3	3	14.62		21	20	3	1	925	35	650	35	0.164	0.021	67
37	$MgAl_2O_4Ca_{12}Al_{14}O_{33}$	5.3	800	650	3	3	14.62		21	90	3	1	925	35	650	35	0.211	0.133	67
38	$Ca_{12}Al_{14}O_{33}$	3	900	640	3	1.5	13	0.85	30	4	2	4	700	40	640	16			68
39	$PdCH_{24}Al_2Mg_6O_{23}$	9.68	800	650	4	2	29.14	0.755	65	1		6	700	20	700	20			69
40	$RhCH_{24}Al_2Mg_6O_{23}$	9.68	800	650	4	2		0.266	65	1		6	700	20	700	20			69
41	$RuCH_{24}Al_2Mg_6O_{23}$	9.68	800	650	4	2		0.957	65	10		6	700	20	700	20			69

Table S10. ARE (%) of Measured vs Predicted CSCM data using GSFrag/ASNN model

The absolute relative error (%) of the measured vs predicted methane conversion and last cycle capacity for unseen CSCM data using the final selected prediction model of ASNN and GSFrag descriptors

N	Methane Conversion (%) {measured}	Methane Conversion (%) {predicted}	ARE (%)	Last cycle CO_2 capacity (gCO_2/gSorbent) {measured}	Last cycle CO_2 capacity (gCO_2/gSorbent) {predicted}	ARE (%)
**5**	0.9352	0.918	1.84			
**1**				0.1146	0.184	60.56
**21**	0.96	0.959	0.10			
**2**				0.060649	0.184	203.39
**23**				0.3967	0.454	14.44
**29**	0.972245	0.997	2.55			
**38**				0.037431	-0.0504	234.65
**24**				0.456	0.474	3.95
**43**	0.2305	0.965	318.66			
**22**				0.2332	0.385	65.09
**27**				0.5257	0.525	0.13
**46**	0.9956	0.755	24.17			
**35**	0.937	0.797	14.94			
**11**				0.09284	0.278	199.44
**10**	0.964	0.73	24.27			
**12**				0.099	0.184	85.86
**3**	0.930637	0.495	46.81			
**13**				0.1921	0.184	4.22
**11**	0.358614	0.802	123.64			
**14**				0.263876	0.184	30.27
**14**	0.224135	0.819	265.40			
**15**				0.280825	0.184	34.48
**31**	0.88621	0.996	12.39			
**7**	0.119	0.63	429.41			
**17**				0.126654	0.184	45.28
**2**	0.825943	0.849	2.79			
**18**				0.3554	0.287	19.25
**18**	0.96	0.96	0.00			
**19**				0.086673	0.184	112.29
**44**	0.2663	0.965	262.37			
**28**				0.3761	0.43	14.33
**6**	0.101	0.535	429.70			
**22**				0.063326	0.184	190.56
**45**	0.3513	0.965	174.69			
**13**	0.588014	0.815	38.60			
**24**				0.271215	0.184	32.16

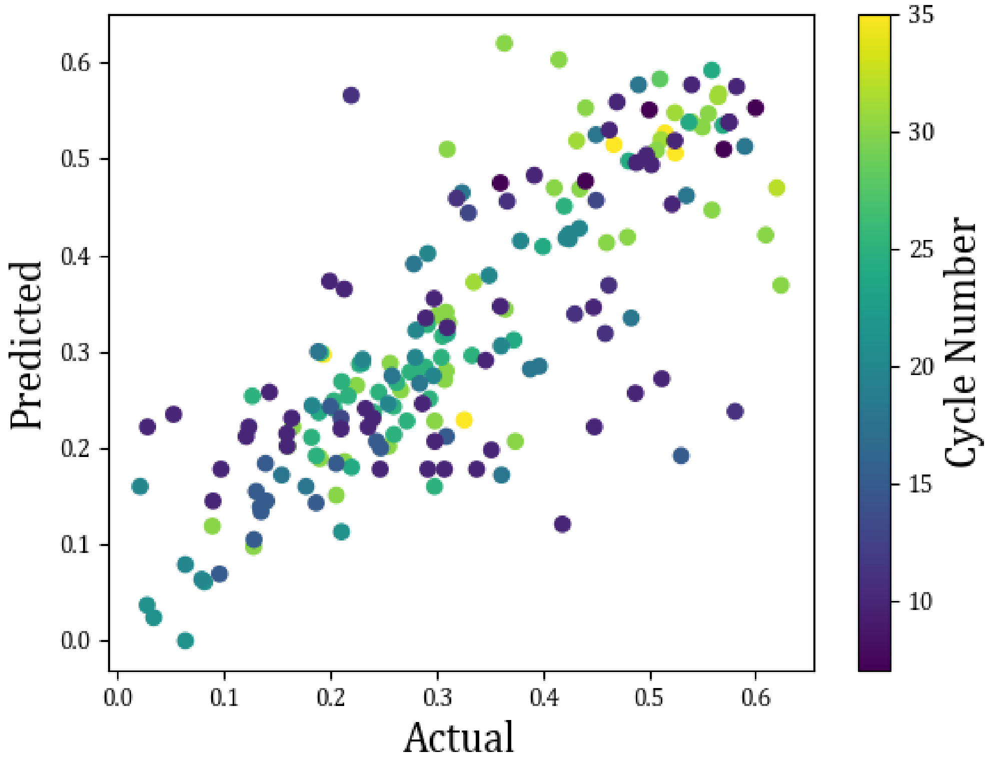

Figure S1. Actual vs Predicted scatter plot showing the effect of the cycle number on the Last cycle capacity (g/g)

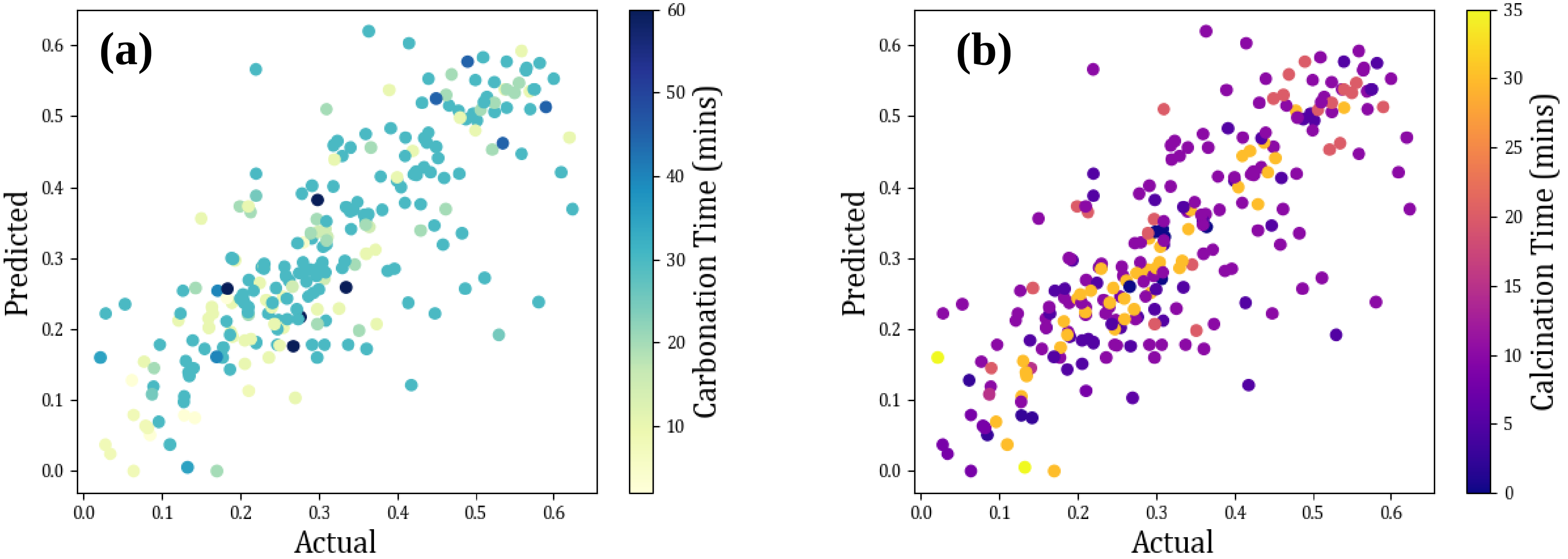

Figure S2. Actual vs Predicted scatter plot showing the effect of the (a) carbonation time and (b) calcination time on the Last cycle capacity (g/g)

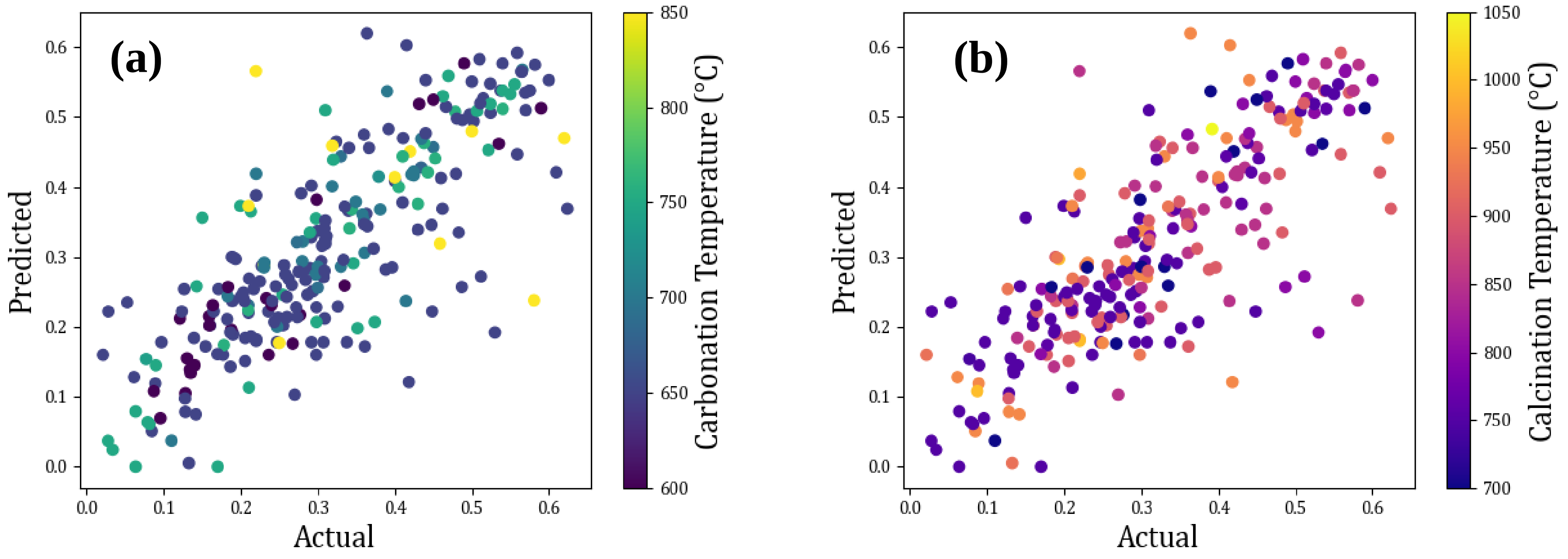

Figure S3. Actual vs Predicted scatter plot showing the effect of the (a) carbonation temperature and (b) calcination temperature on the Last cycle capacity (g/g)

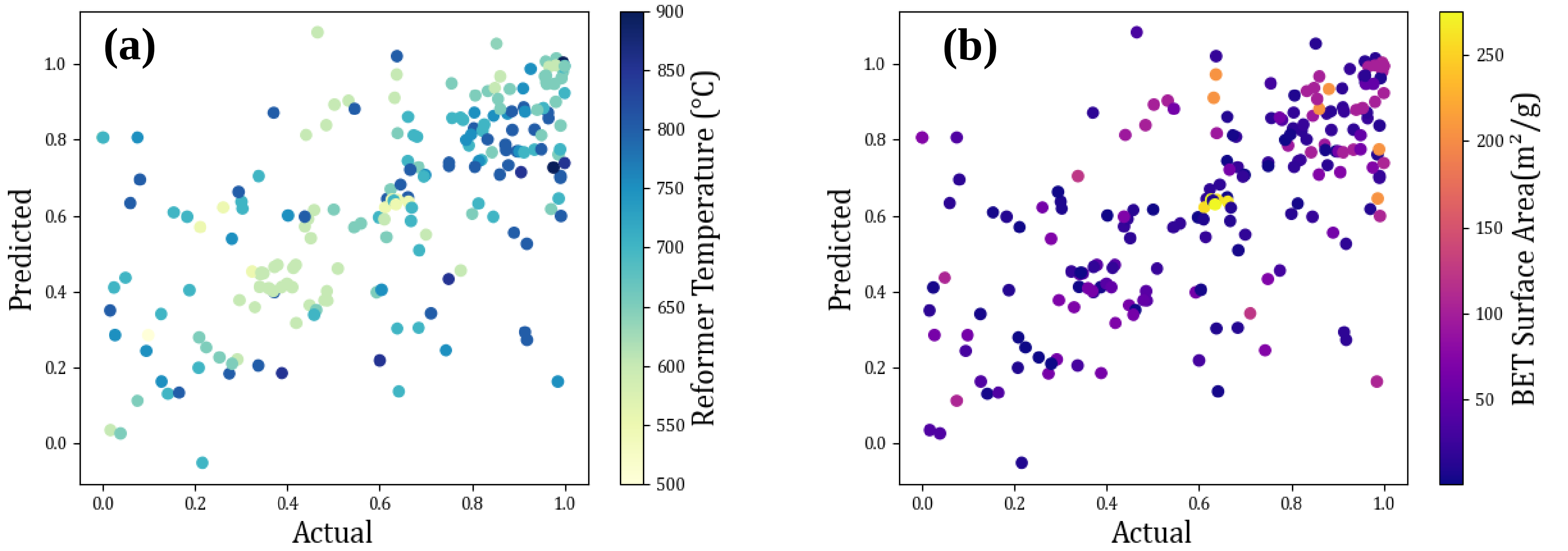

Figure S4. Actual vs Predicted scatter plot showing the effect of the (a) SMR reformer temperature (ºC) and (b) BET Surface Area (m^2/g) on the methane conversion (%)

Database References

(1) Jaworska, J.; Nikolova-Jeliazkova, N.; Aldenberg, T. QSAR Applicability Domain Estimation by Projection of the Training Set in Descriptor Space: A Review. *ATLA Alternatives to Laboratory Animals* **2005**, *33* (5), 445–459. https://doi.org/10.1177/026119290503300508.

(2) Broda, M.; Kierzkowska, A. M.; Müller, C. R. Application of the Sol-Gel Technique to Develop Synthetic Calcium-Based Sorbents with Excellent Carbon Dioxide Capture Characteristics. *ChemSusChem* **2012**, *5* (2), 411–418. https://doi.org/10.1002/cssc.201100468.

(3) Broda, M.; Müller, C. R. Synthesis of Highly Efficient, Ca-Based, Al_2O_3-Stabilized, Carbon Gel-Templated CO_2 Sorbents. *Advanced Materials* **2012**, *24* (22), 3059–3064. https://doi.org/10.1002/adma.201104787.

(4) Zhou, Z.; Qi, Y.; Xie, M.; Cheng, Z.; Yuan, W. Synthesis of CaO-Based Sorbents through Incorporation of Alumina/Aluminate and Their CO_2 Capture Performance. *Chemical Engineering Science* **2012**, *74*, 172–180. https://doi.org/10.1016/j.ces.2012.02.042.

(5) Koirala, R.; Reddy, G. K.; Smirniotis, P. G. Single Nozzle Flame-Made Highly Durable Metal Doped Ca-Based Sorbents for CO_2 Capture at High Temperature. *Energy and Fuels* **2012**, *26* (5), 3103–3109. https://doi.org/10.1021/ef3004015.

(6) Wang, K.; Guo, X.; Zhao, P.; Zheng, C. Cyclic CO_2 Capture of CaO-Based Sorbent in the Presence of Metakaolin and Aluminum (Hydr)Oxides. *Applied Clay Science* **2010**, *50* (1), 41–46. https://doi.org/10.1016/j.clay.2010.06.021.

(7) Manovic, V.; Anthony, E. J. CaO-Based Pellets Supported by Calcium Aluminate Cements for High-Temperature CO_2 Capture. *Environmental Science & Technology* **2009**, *43* (18), 7117–7122. https://doi.org/10.1021/es901258w.

(8) Li, Z.; Cai, N.; Huang, Y.; Han, H. Synthesis, Experimental Studies, and Analysis of a New Calcium-Based Carbon Dioxide Absorbent. *Energy & Fuels* **2005**, *19* (4), 1447–1452. https://doi.org/10.1021/ef0496799.

(9) Wu, S. F.; Li, Q. H.; Kim, J. N.; Yi, K. B. Properties of a Nano CaO/Al_2O_3 CO_2 Sorbent. *Industrial & Engineering Chemistry Research* **2008**, *47* (1), 180–184. https://doi.org/10.1021/ie0704748.

(10) Broda, M.; Kierzkowska, A.; Müller, C. Development of Synthetic, Ca-Based Sorbents for CO_2 Capture Using the Co-Precipitation Technique. *Materials Science* **2011**.

(11) Luo, C.; Zheng, Y.; Ding, N.; Zheng, C. Enhanced Cyclic Stability of CO_2 Adsorption Capacity of CaO-Based Sorbents Using La_2O_3 or $Ca_{12}Al_{14}O_{33}$ as Additives. *Korean Journal of Chemical Engineering* **2011**, *28* (4), 1042–1046. https://doi.org/10.1007/s11814-010-0469-z.

(12) Pacciani, R.; Müller, C. R.; Davidson, J. F.; Dennis, J. S.; Hayhurst, A. N. Synthetic Ca-Based Solid Sorbents Suitable for Capturing CO_2 in a Fluidized Bed. *The Canadian Journal of Chemical Engineering* **2008**, *86* (3), 356–366. https://doi.org/10.1002/cjce.20060.

(13) Stendardo, S.; Andersen, L. K.; Herce, C. Self-Activation and Effect of Regeneration Conditions in CO_2–Carbonate Looping with CaO–$Ca_{12}Al_{14}O_{33}$ Sorbent. *Chemical Engineering Journal* **2013**, *220*, 383–394. https://doi.org/10.1016/j.cej.2013.01.045.

(14) Martavaltzi, C. S.; Lemonidou, A. A. Parametric Study of the CaO-$Ca_{12}Al_{14}O_{33}$ Synthesis with Respect to High CO_2 Sorption Capacity and Stability on Multicycle Operation. *Industrial and Engineering Chemistry Research* **2008**, *47* (23), 9537–9543. https://doi.org/10.1021/ie800882d.

(15) Martavaltzi, C. S.; Lemonidou, A. A. Development of New CaO Based Sorbent Materials for CO_2 Removal at High Temperature. *Microporous and Mesoporous Materials* **2008**, *110* (1), 119–127. https://doi.org/10.1016/j.micromeso.2007.10.006.

(16) Wu, S. F.; Zhu, Y. Q. Behavior of $CaTiO_3$ /Nano-CaO as a CO_2 Reactive Adsorbent. *Industrial & Engineering Chemistry Research* **2010**, *49* (6), 2701–2706. https://doi.org/10.1021/ie900900r.

(17) Zhao, M.; Bilton, M.; Brown, A. P.; Cunliffe, A. M.; Dvininov, E.; Dupont, V.; Comyn, T. P.; Milne, S. J. Durability of CaO–$CaZrO_3$ Sorbents for High-Temperature CO_2 Capture Prepared by a Wet Chemical Method. *Energy & Fuels* **2014**, *28* (2), 1275–1283. https://doi.org/10.1021/ef4020845.

(18) Wang, S.; Fan, S.; Fan, L.; Zhao, Y.; Ma, X. Effect of Cerium Oxide Doping on the Performance of CaO-Based Sorbents during Calcium Looping Cycles. *Environmental Science & Technology* **2015**, *49* (8), 5021–5027. https://doi.org/10.1021/es5052843.

(19) Li, L.; King, D. L.; Nie, Z.; Howard, C. Magnesia-Stabilized Calcium Oxide Absorbents with Improved Durability for High Temperature CO_2 Capture. *Industrial & Engineering Chemistry Research* **2009**, *48* (23), 10604–10613. https://doi.org/10.1021/ie901166b.

(20) Lan, P.; Wu, S. Synthesis of a Porous Nano-CaO/MgO-Based CO_2 Adsorbent. *Chemical Engineering & Technology* **2014**, *37* (4), 580–586. https://doi.org/10.1002/ceat.201300709.

(21) Albrecht, K. O.; Wagenbach, K. S.; Satrio, J. A.; Shanks, B. H.; Wheelock, T. D. Development of a CaO-Based CO_2 Sorbent with Improved Cyclic Stability. *Industrial & Engineering Chemistry Research* **2008**, *47* (20), 7841–7848. https://doi.org/10.1021/ie8007743.

(22) Zhao, M.; Yang, X.; Church, T. L.; Harris, A. T. Novel CaO–SiO_2 Sorbent and Bifunctional Ni/Co–CaO/SiO_2 Complex for Selective H_2 Synthesis from Cellulose. *Environmental Science & Technology* **2012**, *46* (5), 2976–2983. https://doi.org/10.1021/es300135d.

(23) Derevschikov, V. S.; Lysikov, A. I.; Okunev, A. G. High Temperature CaO/Y_2O_3 Carbon Dioxide Absorbent with Enhanced Stability for Sorption-Enhanced Reforming Applications. *Industrial & Engineering Chemistry Research* **2011**, *50* (22), 12741–12749. https://doi.org/10.1021/ie2015334.

(24) Zhang, X.; Li, Z.; Peng, Y.; Su, W.; Sun, X.; Li, J. Investigation on a Novel CaO–Y_2O_3 Sorbent for Efficient CO_2 Mitigation. *Chemical Engineering Journal* **2014**, *243*, 297–304. https://doi.org/10.1016/j.cej.2014.01.017.

(25) Hu, Y.; Liu, W.; Chen, H.; Zhou, Z.; Wang, W.; Sun, J.; Yang, X.; Li, X.; Xu, M. Screening of Inert Solid Supports for CaO-Based Sorbents for High Temperature CO_2 Capture. *Fuel* **2016**, *181*, 199–206. https://doi.org/10.1016/j.fuel.2016.04.138.

(26) Radfarnia, H. R.; Iliuta, M. C. Metal Oxide-Stabilized Calcium Oxide CO_2 Sorbent for Multicycle Operation. *Chemical Engineering Journal* **2013**, *232*, 280–289. https://doi.org/10.1016/j.cej.2013.07.049.

(27) Lu, H.; Khan, A.; Pratsinis, S. E.; Smirniotis, P. G. Flame-Made Durable Doped-CaO Nanosorbents for CO_2 Capture. *Energy & Fuels* **2009**, *23* (2), 1093–1100. https://doi.org/10.1021/ef8007882.

(28) Radfarnia, H. R.; Iliuta, M. C. Development of Zirconium-Stabilized Calcium Oxide Absorbent for Cyclic High-Temperature CO_2 Capture. *Industrial & Engineering Chemistry Research* **2012**, *51* (31), 10390–10398. https://doi.org/10.1021/ie301287k.

(29) Li, L.; King, D. L.; Nie, Z.; Li, X. S.; Howard, C. $MgAl_2O_4$ Spinel-Stabilized Calcium Oxide Absorbents with Improved Durability for High-Temperature CO_2 Capture. *Energy & Fuels* **2010**, *24* (6), 3698–3703. https://doi.org/10.1021/ef100245q.

(30) Zhang, M.; Peng, Y.; Sun, Y.; Li, P.; Yu, J. Preparation of CaO-Al_2O_3 Sorbent and CO_2 Capture Performance at High Temperature. *Fuel* **2013**, *111*, 636–642. https://doi.org/10.1016/j.fuel.2013.03.078.

(31) Li, Y.; Su, M.; Xie, X.; Wu, S.; Liu, C. CO_2 Capture Performance of Synthetic Sorbent Prepared from Carbide Slag and Aluminum Nitrate Hydrate by Combustion Synthesis. *Applied Energy* **2015**, *145*, 60–68. https://doi.org/10.1016/j.apenergy.2015.01.061.

(32) Liu, F. Q.; Li, W. H.; Liu, B. C.; Li, R. X. Synthesis, Characterization, and High Temperature CO_2 Capture of New CaO Based Hollow Sphere Sorbents. *Journal of Materials Chemistry A* **2013**, *1* (27), 8037–8044. https://doi.org/10.1039/c3ta11369h.

(33) Xu, P.; Xie, M.; Cheng, Z.; Zhou, Z. CO_2 Capture Performance of CaO-Based Sorbents Prepared by a Sol-Gel Method. *Industrial and Engineering Chemistry Research* **2013**, *52* (34), 12161–12169. https://doi.org/10.1021/ie401600e.

(34) Liu, W.; Feng, B.; Wu, Y.; Wang, G.; Barry, J.; Diniz da Costa, J. C. Synthesis of Sintering-Resistant Sorbents for CO_2 Capture. *Environmental Science & Technology* **2010**, *44* (8), 3093–3097. https://doi.org/10.1021/es903436v.

(35) Radfarnia, H. R.; Sayari, A. A Highly Efficient CaO-Based CO_2 Sorbent Prepared by a Citrate-Assisted Sol–Gel Technique. *Chemical Engineering Journal* **2015**, *262*, 913–920. https://doi.org/10.1016/j.cej.2014.09.074.

(36) Florin, N. H.; Blamey, J.; Fennell, P. S. Synthetic CaO-Based Sorbent for CO_2 Capture from Large-Point Sources. *Energy and Fuels* **2010**, *24* (8), 4598–4604. https://doi.org/10.1021/ef100447c.

(37) Qin, C.; Liu, W.; An, H.; Yin, J.; Feng, B. Fabrication of CaO-Based Sorbents for CO_2 Capture by a Mixing Method. *Environmental Science & Technology* **2012**, *46* (3), 1932–1939. https://doi.org/10.1021/es203525y.

(38) Ali, S.; Al-Marri, M. J.; Abdelmoneim, A. G.; Kumar, A.; Khader, M. M. Catalytic Evaluation of Nickel Nanoparticles in Methane Steam Reforming. *International Journal of Hydrogen Energy* **2016**, *41* (48), 22876–22885. https://doi.org/10.1016/j.ijhydene.2016.08.200.

(39) Katheria, S.; Gupta, A.; Deo, G.; Kunzru, D. Effect of Calcination Temperature on Stability and Activity of Ni/$MgAl_2O_4$ Catalyst for Steam Reforming of Methane at High Pressure Condition. *International Journal of Hydrogen Energy* **2016**, *41* (32), 14123–14132. https://doi.org/10.1016/j.ijhydene.2016.05.109.

(40) Rogers, J. L.; Mangarella, M. C.; D'Amico, A. D.; Gallagher, J. R.; Dutzer, M. R.; Stavitski, E.; Miller, J. T.; Sievers, C. Differences in the Nature of Active Sites for Methane Dry Reforming and Methane Steam Reforming over Nickel Aluminate Catalysts. *ACS Catalysis* **2016**, *6* (9), 5873–5886. https://doi.org/10.1021/acscatal.6b01133.

(41) Khani, Y.; Shariatinia, Z.; Bahadoran, F. High Catalytic Activity and Stability of $ZnLaAlO_4$ Supported Ni, Pt and Ru Nanocatalysts Applied in the Dry, Steam and Combined Dry-Steam Reforming of Methane. *Chemical Engineering Journal* **2016**, *299*, 353–366. https://doi.org/10.1016/j.cej.2016.04.108.

(42) Fang, X.; Zhang, X.; Guo, Y.; Chen, M.; Liu, W.; Xu, X.; Peng, H.; Gao, Z.; Wang, X.; Li, C. Highly Active and Stable Ni/$Y_2Zr_2O_7$ Catalysts for Methane Steam Reforming: On the Nature and Effective

Preparation Method of the Pyrochlore Support. *International Journal of Hydrogen Energy* **2016**, *41* (26), 11141–11153. https://doi.org/10.1016/j.ijhydene.2016.04.038.

(43) Yoo, J.; Park, S.; Song, J. H.; Yoo, S.; Song, I. K. Hydrogen Production by Steam Reforming of Natural Gas over Butyric Acid-Assisted Nickel/Alumina Catalyst. *International Journal of Hydrogen Energy* **2017**, *42* (47), 28377–28385. https://doi.org/10.1016/j.ijhydene.2017.09.148.

(44) Zhang, X.; Peng, L.; Fang, X.; Cheng, Q.; Liu, W.; Peng, H.; Gao, Z.; Zhou, W.; Wang, X. $Ni/Y_2B_2O_7$ (B=Ti, Sn, Zr and Ce) Catalysts for Methane Steam Reforming: On the Effects of B Site Replacement. *International Journal of Hydrogen Energy* **2018**, *43* (17), 8298–8312. https://doi.org/10.1016/j.ijhydene.2018.03.086.

(45) Fang, X.; Xu, L.; Zhang, X.; Zhang, K.; Dai, H.; Liu, W.; Xu, X.; Wang, X.; Zhou, W. Effect of Rare Earth Element (Ln = La, Pr, Sm, and Y) on Physicochemical Properties of the $Ni/Ln_2Ti_2O_7$ Catalysts for the Steam Reforming of Methane. *Molecular Catalysis* **2019**, *468*, 130–138. https://doi.org/10.1016/j.mcat.2019.02.022.

(46) Dan, M.; Mihet, M.; Lazar, M. D. Hydrogen and/or Syngas Production by Combined Steam and Dry Reforming of Methane on Nickel Catalysts. *International Journal of Hydrogen Energy* **2020**, *45* (49), 26254–26264. https://doi.org/10.1016/j.ijhydene.2019.12.158.

(47) Lertwittayanon, K.; Youravong, W.; Lau, W. J. Enhanced Catalytic Performance of $Ni/\alpha\text{-}Al_2O_3$ Catalyst Modified with $CaZrO_3$ Nanoparticles in Steam-Methane Reforming. *International Journal of Hydrogen Energy* **2017**, *42* (47), 28254–28265. https://doi.org/10.1016/j.ijhydene.2017.09.030.

(48) Azancot, L.; Bobadilla, L. F.; Santos, J. L.; Córdoba, J. M.; Centeno, M. A.; Odriozola, J. A. Influence of the Preparation Method in the Metal-Support Interaction and Reducibility of Ni-Mg-Al Based Catalysts for Methane Steam Reforming. *International Journal of Hydrogen Energy* **2019**, *44* (36), 19827–19840. https://doi.org/10.1016/j.ijhydene.2019.05.167.

(49) Kusakabe, K.; Sotowa, K. I.; Eda, T.; Iwamoto, Y. Methane Steam Reforming over $Ce\text{-}ZrO_2$-Supported Noble Metal Catalysts at Low Temperature. *Fuel Processing Technology* **2004**, *86* (3), 319–326. https://doi.org/10.1016/j.fuproc.2004.05.003.

(50) Urasaki, K.; Sekine, Y.; Kawabe, S.; Kikuchi, E.; Matsukata, M. Catalytic Activities and Coking Resistance of Ni/Perovskites in Steam Reforming of Methane. *Applied Catalysis A: General* **2005**, *286* (1), 23–29. https://doi.org/10.1016/j.apcata.2005.02.020.

(51) Jeong, J. H.; Lee, J. W.; Seo, D. J.; Seo, Y.; Yoon, W. L.; Lee, D. K.; Kim, D. H. Ru-Doped Ni Catalysts Effective for the Steam Reforming of Methane without the Pre-Reduction Treatment with H_2. *Applied Catalysis A: General* **2006**, *302* (2), 151–156. https://doi.org/10.1016/j.apcata.2005.12.007.

(52) Xu, P.; Zhou, Z.; Zhao, C.; Cheng, Z. Catalytic Performance of $Ni/CaO\text{-}Ca_5Al_6O_{14}$ Bifunctional Catalyst Extrudate in Sorption-Enhanced Steam Methane Reforming. *Catalysis Today* **2016**, *259*, 347–353. https://doi.org/10.1016/j.cattod.2015.05.026.

(53) Zhao, C.; Zhou, Z.; Cheng, Z.; Fang, X. Sol-Gel-Derived, $CaZrO_3$-Stabilized $Ni/CaO\text{-}CaZrO_3$ Bifunctional Catalyst for Sorption-Enhanced Steam Methane Reforming. *Applied Catalysis B: Environmental* **2016**, *196*, 16–26. https://doi.org/10.1016/j.apcatb.2016.05.021.

(54) Vanga, G.; Gattia, D. M.; Stendardo, S.; Scaccia, S. Novel Synthesis of Combined $CaO\text{-}Ca_{12}Al_{14}O_{33}$-Ni Sorbent-Catalyst Material for Sorption Enhanced Steam Reforming Processes. *Ceramics International* **2019**, *45* (6), 7594–7605. https://doi.org/10.1016/j.ceramint.2019.01.054.

(55) di Giuliano, A.; Gallucci, K.; Kazi, S. S.; Giancaterino, F.; di Carlo, A.; Courson, C.; Meyer, J.; di Felice, L. Development of Ni- and CaO-Based Mono- and Bi-Functional Catalyst and Sorbent

Materials for Sorption Enhanced Steam Methane Reforming: Performance over 200 cycles and Attrition Tests. *Fuel Processing Technology* **2019**, *195*. https://doi.org/10.1016/j.fuproc.2019.106160.

(56) Chen, X.; Yang, L.; Zhou, Z.; Cheng, Z. Core-Shell Structured CaO-$Ca_9Al_6O_{18}$@$Ca_5Al_6O_{14}$/Ni Bifunctional Material for Sorption-Enhanced Steam Methane Reforming. *Chemical Engineering Science* **2017**, *163*, 114–122. https://doi.org/10.1016/j.ces.2017.01.036.

(57) di Giuliano, A.; Girr, J.; Massacesi, R.; Gallucci, K.; Courson, C. Sorption Enhanced Steam Methane Reforming by Ni–CaO Materials Supported on Mayenite. *International Journal of Hydrogen Energy* **2017**, *42* (19), 13661–13680. https://doi.org/10.1016/j.ijhydene.2016.11.198.

(58) Cesário, M. R.; Barros, B. S.; Courson, C.; Melo, D. M. A.; Kiennemann, A. Catalytic Performances of Ni-CaO-Mayenite in CO_2 Sorption Enhanced Steam Methane Reforming. *Fuel Processing Technology* **2015**, *131*, 247–253. https://doi.org/10.1016/j.fuproc.2014.11.028.

(59) di Giuliano, A.; Gallucci, K.; di Carlo, A.; Stendardo, S.; Courson, C.; Foscolo, P. U. Sorption Enhanced Steam Methane Reforming by Ni/CaO/Mayenite Combined Systems: Overview of Experimental Results from European Research Project ASCENT. *Canadian Journal of Chemical Engineering* **2020**, *98* (9), 1907–1923. https://doi.org/10.1002/cjce.23779.

(60) Martavaltzi, C. S.; Lemonidou, A. A. Hydrogen Production via Sorption Enhanced Reforming of Methane: Development of a Novel Hybrid Material-Reforming Catalyst and CO_2 Sorbent. *Chemical Engineering Science* **2010**, *65* (14), 4134–4140. https://doi.org/10.1016/j.ces.2010.04.038.

(61) Phromprasit, J.; Powell, J.; Wongsakulphasatch, S.; Kiatkittipong, W.; Bumroongsakulsawat, P.; Assabumrungrat, S. H_2 Production from Sorption Enhanced Steam Reforming of Biogas Using Multifunctional Catalysts of Ni over Zr-, Ce- and La-Modified CaO Sorbents. *Chemical Engineering Journal* **2017**, *313*, 1415–1425. https://doi.org/10.1016/j.cej.2016.11.051.

(62) Aloisi, I.; di Giuliano, A.; di Carlo, A.; Foscolo, P. U.; Courson, C.; Gallucci, K. Sorption Enhanced Catalytic Steam Methane Reforming: Experimental Data and Simulations Describing the Behaviour of Bi-Functional Particles. *Chemical Engineering Journal* **2017**, *314*, 570–582. https://doi.org/10.1016/j.cej.2016.12.014.

(63) Xu, P.; Zhou, Z. M.; Zhao, C.; Cheng, Z. Ni/CaO-Al_2O_3 Bifunctional Catalysts for Sorption-Enhanced Steam Methane Reforming. *AIChE Journal* **2014**, *60* (10), 3547–3556. https://doi.org/10.1002/aic.14543.

(64) Radfarnia, H. R.; Iliuta, M. C. Hydrogen Production by Sorption-Enhanced Steam Methane Reforming Process Using CaO-Zr/Ni Bifunctional Sorbent-Catalyst. *Chemical Engineering and Processing: Process Intensification* **2014**, *86*, 96–103. https://doi.org/10.1016/j.cep.2014.10.014.

(65) Radfarnia, H. R.; Iliuta, M. C. Development of Al-Stabilized CaO-Nickel Hybrid Sorbent-Catalyst for Sorption-Enhanced Steam Methane Reforming. *Chemical Engineering Science* **2014**, *109*, 212–219. https://doi.org/10.1016/j.ces.2014.01.033.

(66) Feng, H. Z.; Lan, P. Q.; Wu, S. F. A Study on the Stability of a NiO-CaO/Al_2O_3 Complex Catalyst by La_2O_3 Modification for Hydrogen Production. *International Journal of Hydrogen Energy* **2012**, *37* (19), 14161–14166. https://doi.org/10.1016/j.ijhydene.2012.06.099.

(67) di Felice, L.; Kazi, S. S.; Sørby, M. H.; Martinez, I.; Grasa, G.; Maury, D.; Meyer, J. Combined Sorbent and Catalyst Material for Sorption Enhanced Reforming of Methane under Cyclic Regeneration in Presence of H_2O and CO_2. *Fuel Processing Technology* **2019**, *183*, 35–47. https://doi.org/10.1016/j.fuproc.2018.10.012.

(68) Micheli, F.; Sciarra, M.; Courson, C.; Gallucci, K. Catalytic Steam Methane Reforming Enhanced by CO_2 Capture on CaO Based Bi-Functional Compounds. *Journal of Energy Chemistry* **2017**, *26* (5), 1014–1025. https://doi.org/10.1016/j.jechem.2017.09.001.

(69) Dang, C.; Long, J.; Li, H.; Cai, W.; Yu, H. Pd-Promoted Ni-Ca-Al Bi-Functional Catalyst for Integrated Sorption-Enhanced Steam Reforming of Glycerol and Methane Reforming of Carbonate. *Chemical Engineering Science* **2021**, *230*. https://doi.org/10.1016/j.ces.2020.116226.
